# Less Is More: A Physiological Dose of Vitamin B12 Enhances Neural Recovery Compared to a High Dose in an H₂O₂-Stressed SH-SY5Y Neural-Like Cell Model

**DOI:** 10.1007/s12035-026-05841-9

**Published:** 2026-04-17

**Authors:** Aimee Rachel Mathew, Luca Buccini, Anacleto Proietti, Giacomo Di Matteo, Erisa Selita, Ilaria Serangeli, Roberta Stefanelli, Elisa Gazzera, Francesco Mura, Luisa Mannina, Marco Rossi, Antonella De Jaco, Elena Miranda, Ada Maria Tata, Livia Angeloni, Piergiorgio La Rosa, Daniele Passeri, Virve Cavallucci, Marco Fidaleo

**Affiliations:** 1 Department of Biology and Biotechnologies “Charles Darwin”, Sapienza University of Rome, Rome, Italy; 2 Department of Basic and Applied Sciences for Engineering, Sapienza University of Rome, Rome, Italy; 3 Research Centre for Nanotechnology for Engineering of Sapienza (CNIS), Sapienza University of Rome, Rome, Italy; 4 Department of Chemistry and Technology of Drugs, Sapienza University of Rome, Rome, Italy; 5 NMR-Based Metabolomics Laboratory (NMLab), Sapienza University of Rome, Rome, Italy; 6 Fondazione Policlinico Universitario A. Gemelli IRCCS, Rome, Italy; 7 Department of Psychology, Division of Neuroscience, Sapienza University of Rome, Rome, Italy; 8 Department of Neuroscience, Section of Human Anatomy, Catholic University of the Sacred Heart, Rome, Italy; 9 Dipartimento di Medicina e Chirurgia Traslazionale, Università Cattolica del Sacro Cuore, Rome, Italy

**Keywords:** Vitamin B12, Oxidative damage, Neural recovery, Mitochondrial activity, Lipid remodeling, Neurite outgrowth, SH-SY5Y model

## Abstract

Vitamin B12 (VitB12) plays a crucial role in neural homeostasis, and a high dose is preferred as treatment to support recovery from neural impairments. However, a significant knowledge gap remains regarding its dose-dependent effects, particularly in its influence on cellular recovery following neural damage. To address this, we utilized retinoic acid-differentiated SH-SY5Y cells, as an in vitro neural model, where we induced neural damage using hydrogen peroxide (H_2_O_2_), followed by recovery in either a physiological or high dose of VitB12. Our findings reveal that a physiological dose of VitB12 promotes more efficient recovery by enhancing cell survival and promoting neurite elongation after H_2_O_2_ insult, compared to a high dose. Recovery with a physiological dose of VitB12 was associated with an early-stage (2 h) activation of antioxidant defenses, suggesting a quicker cellular response to oxidative stress than the high dose. At later stages (24 h), recovery with a physiological dose of VitB12 enhances mitochondrial metabolic activity and morphodynamics, alongside promoting lipid remodeling and increased formation of lipid droplets (LDs). These lipid-related processes may collectively contribute to mitigating oxidative damage and reinforcing cellular resilience during recovery. Overall, our study highlights that a physiological dose of VitB12 not only activates antioxidant defenses earlier than the high dose but also induces later-stage lipid remodeling, potentially supporting neural recovery and homeostasis.

**Graphical Abstract:**

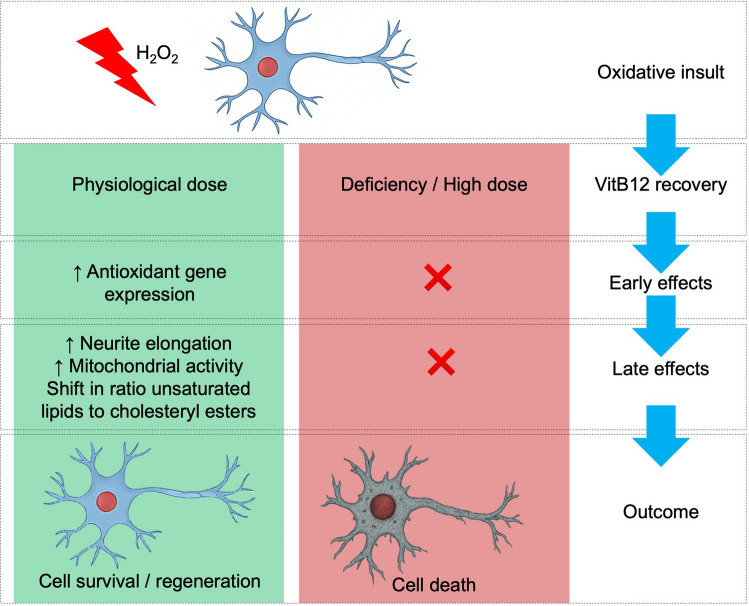

**Supplementary Information:**

The online version contains supplementary material available at 10.1007/s12035-026-05841-9.

## Introduction

Vitamin B12 (VitB12) has been recognized, since the 1960 s, as an indispensable cofactor in vital metabolic pathways and essential for maintaining cellular homeostasis. The detrimental effects of VitB12 deficiency have been extensively studied, demonstrating its contribution to multi-organ damage, including impaired nervous system function [1–4]. Epidemiologically, determining the exact prevalence of VitB12 deficiency is difficult due to multiple factors. In older adults, age-related organ dysfunction leads to deficiency rates between 1.2 and 40%, varying with living conditions, illness, and nutritional status [5–9].

Recent studies have highlighted a potential role of VitB12 in promoting neural recovery processes, following injury or impairment. For instance, VitB12 has been shown to enhance dendritic branching, improve synaptic plasticity, and boost memory performance in elderly mice [10]. It has also been found to counteract neuronal damage caused by endoplasmic reticulum (ER) stress and support the repair of injured nerve tissue [11]. Furthermore, VitB12 supplementation has demonstrated the ability to reverse the decline in dendritic complexity and spine density associated with traumatic brain injury (TBI), leading to improved synaptic plasticity, increased neuronal survival, and the restoration of cognitive functions [12]. In vitro experiments on SH-SY5Y cells, a widely used model for neural-like behavior studies, have likewise demonstrated the neuroprotective effects of VitB12 against H_2_O_2_-induced neuronal damage, mediated through both transcriptomic modulation [13] and lipidomic modification [14].

Collectively, the results of the mentioned studies suggest a potentially critical role for VitB12 in facilitating neuronal recovery after damage. Although these studies directly address the role of VitB12 in neural repair, significant gaps remain in our understanding of the precise biological mechanisms involved.

At the biochemical level, VitB12 plays a crucial role in both cytoplasmic and mitochondrial metabolic pathways. It is involved in the remethylation of homocysteine to methionine via methionine synthase (MTR) [1–4] and in the conversion of L-methylmalonyl-CoA to succinyl-CoA through L-methylmalonyl-CoA mutase (MUT) [1, 3]. Consequently, researchers have sought to link neurological dysfunctions with metabolic disturbances. However, recent studies have revealed that the consequences of VitB12 deficiency extend beyond the accumulation of toxic intermediates or substrate deficiencies (metabolic decompensation). They also include more profound cellular alterations, including molecular and structural changes at both the cellular and tissue levels (reviewed in [15]).

High-dose VitB12 therapy is the preferred treatment approach, as it is generally considered non-toxic, with a low risk of severe adverse effects even with prolonged administration (reviewed in [16]). Nonetheless, dermatological reactions have been reported in some cases [17]. Although high-dose VitB12 is widely used and demonstrates therapeutic efficacy, a review of studies on VitB12 treatment highlights a significant challenge in determining the optimal dosage for achieving maximum therapeutic benefit [15]. To clarify the potential role of VitB12 dosage in neuroprotection and explore its underlying mechanisms, we present a study investigating dose-dependent effects in SH-SY5Y neuronal cells exposed to oxidative stress induced by hydrogen peroxide (H_2_O_2_), a model that simulates neuronal damage [18, 19]. The results of this in vitro study showed that supplementation with a physiological dose of VitB12 significantly improved cell survival, promoted neural outgrowth, and rapidly upregulated genes linked to antioxidant defense within a few hours, providing a faster and more efficient response to H_2_O_2_-induced injury compared to the higher dose. Furthermore, the enhancement of mitochondrial network fusion, the relative enrichment of unsaturated lipids, and the increased formation of lipid droplets (LDs) observed at later time points may represent a delayed protective mechanism that is not evident with the higher dose. Overall, these findings suggest that a physiological dose of VitB12 is more effective in promoting cellular recovery from H_2_O_2_-induced damage.

## Materials and Methods

### Cell Culture

Human neuroblastoma cell line SH-SY5Y were cultured in flasks and dishes and maintained in MEM (Aurogene) supplemented with 10% FBS (Sigma-Aldrich), 1% streptomycin (Cytiva), 50 IU/mL penicillin (Cytiva), 1% L-glutamine (Cytiva), 1% amphotericin (Cytiva), and 0.2% normocin (InvivoGen) in a humidified atmosphere of 5% CO_2_ in air at 37 °C inside an incubator. The medium for cell culture was replaced every 3 days, and the cells were subcultured on reaching 85–95% confluency. The number of cell passages was always maintained between 2 and 15. All cell culture techniques were performed inside a laminar air flow chamber hood under sterile conditions.

### SH-SY5Y Differentiation and Treatments

For differentiation/treatment experiments, cells were seeded at a density of 8.33 × 10^4^ cells per cm^2^, and for microscopic images and staining experiments, cells were seeded at a density of 4.16 × 10^4^ cells per cm^2^. The seeding was performed 1 day prior to the start of differentiation/treatment experiments. After 24 h of cell seeding, differentiation was induced by decreasing the amount of FBS in the culture medium, followed by adding retinoic acid (RA). RA stock was prepared by dissolving 3 mg of RA powder (Sigma-Aldrich) in 1 mL in absolute ethanol, such that the concentration of the stock is 10 mM. Since RA is extremely light-sensitive, precautions were taken while adding it into the medium in the absence of light. The differentiation was terminated by removing the differentiation media after 3 days (72 h). At the end of 3 days, for oxidatively insulting the differentiated SH-SY5Y cells, an H_2_O_2_ stock of 10 mM was prepared by dissolving 30% H_2_O_2_ (Sigma-Aldrich) solution in ultrapure water. This was further diluted in the MEM medium (with 0% FBS) to prepare the final concentration, and the cells were incubated for 30 min at 37 °C inside an incubator for inducing oxidative stress. It should be ensured that the cells were devoid of any traces of FBS, by rinsing the wells with 1× phosphate-buffered saline (PBS) (Aurogene). For evaluating the role of VitB12 in the neural recovery from the damage induced by the oxidative stress, a stock of 20 mM VitB12 (Sigma-Aldrich) was dissolved in injectable water, followed by dissolving the stock in MEM containing 1% FBS to prepare the final concentration with the physiological and a high dose of VitB12. Because cobalamins are photolabile, VitB12 stock and working solutions were handled under reduced-light conditions and prepared from fresh or single-use aliquots to minimize photodegradation. VitB12-supplemented recovery media were prepared immediately before use. This medium was later added to the cells, and the plates were incubated at 37 °C inside an incubator for neural recovery. It should be ensured that the cells were devoid of any traces of H_2_O_2_ before adding the media containing VitB12, by rinsing the wells with 1× PBS. Furthermore, the images of the differentiated cells under different conditions were captured using CamLabLite camera and software (Bresser MikroCam, Informer Technologies, Inc.) and the average number of neurites per cell and the mean neurite outgrowth length per cell were quantified using the NeuroJ plugin developed for the ImageJ software [20].

### Cell Counting and Erythrosin B–Stained Positive Assay

For cell counting experiments, cells were seeded at a density of 8.33 × 10^4^ cells per cm^2^, 1 day prior to the start of the experiment. Cell counting experiments were performed to investigate the optimum differentiation protocol and optimum concentration of H_2_O_2_ and to identify the extent of cell growth and cell death induced by various treatments with H_2_O_2_ and/or VitB12. After 24 h of cell seeding, differentiation was induced in the SH-SY5Y cells, followed by several treatments with H_2_O_2_ and/or VitB12. After the respective treatments, the media were collected from all the wells, and the wells were washed with 1× PBS, which were also collected. This step was performed in order to collect all the dead cells that were detached and floating in the medium. The live cells in the wells were trypsinized gently using 1× trypsin (Cytiva) and were collected in the same centrifuge tubes, followed by centrifugation at 6000 rpm at 4 °C for 5 min. The cell pellets were resuspended in 1× PBS, and the live and dead cell numbers were obtained from LunaFX7 Automated Cell Counter (Logos Biosystems) using the erythrosin B staining (Logos Biosystems) method by mixing cell suspension and trypan blue in the ratio 1:1.

### Western Blot

For western blot analysis, the cells were seeded at a density of 8.33 × 10^4^ cells per cm^2^. The relative expression of several mature neuronal markers, proteins, and a transcriptional factor was evaluated by western blot immunoassay. After the respective treatments, cells were washed with 1× PBS to remove excess of the culture medium. The cells were lysed directly with western blot sample buffer (6.25% 1 M Tris pH = 6.8, 2% sodium dodecyl sulfate, 10% glycerol, 0.01% bromophenol blue, and 5% β-mercaptoethanol, from Invitrogen/Gibco/Sigma-Aldrich, in distilled water) or lysed with NP-40 lysis buffer (1 M Tris-HCl pH = 8, 5 M NaCl, 10% NP-40 from Sigma-Aldrich in distilled water), added with phosphatase inhibitors—50 µM NaF, 1 µM Na3VO4, 1 µM PMSF, and protease inhibitor cocktail from Sigma-Aldrich—and the lysates were sonicated. Protein content of the extracts was quantified using Bradford assay (Thermo Scientific), and 30 µg of total protein extracts was then separated by sodium dodecyl sulfate-polyacrylamide gel electrophoresis (SDS-PAGE). Based on the molecular weights of the proteins of interest to be assessed, the stacking gel and the resolving gel contained 4% and 8–14% of acrylamide (Sigma-Aldrich), respectively. The separated proteins were then electrotransferred onto a polyvinylidene fluoride (PVDF) membrane (Merck Life Science), previously activated in 20% methanol (Fisher Scientific). The membrane was then incubated for 1 h at room temperature in 5% non-fat dry milk (PanReac AppliChem-ITW Reagents) and 0.2% Tween-20 (Fisher Scientific) in Tris-buffered saline (TBST) for blocking the non-specific bindings. After the blocking step, the membranes were washed with 1× TBST and probed with the primary antibodies in 5% non-fat dry milk or 3% bovine serum albumin (BSA) (Sigma-Aldrich) and 0.05% sodium azide (Sigma-Aldrich) in TBST (to prevent bacterial growth) at 4 °C overnight. The primary antibodies included—proliferative cell nuclear antigen (PCNA) (Santa Cruz Biotechnology), synaptophysin (SYP) (Santa Cruz Biotechnology), growth associated protein-43 (GAP-43) (Santa Cruz Biotechnology), CD320 (AB clonal), caspase-3 (AB clonal), Poly(ADP-ribose) polymerase 1 (PARP1) (AB clonal), catalase (Abcam), and β-actin (Immunological Sciences), diluted as per the instructions from the manufacturer. After removing the primary antibodies and washing the membranes with 1× TBST, the membranes were incubated with the corresponding secondary antibodies (Fortis Life Sciences) (diluted as per the instructions from the manufacturer), which were conjugated with horseradish peroxidase, at room temperature for 1 h. After removing the secondary antibodies, the membranes were washed with 1× TBST, and the bands were visualized by enhanced chemiluminescence assay (HyGLO, Denville Scientific Inc.) using ChemiDoc XRS + (BIO-RAD) and Image Lab software (BIO-RAD).

### RNA Extraction and qPCR

Total RNA was isolated utilizing the Total RNA Purification Plus Kit (Norgen Biotek Corp.) in accordance with the manufacturer’s guidelines. Then, 1 µg of RNA was reverse-transcribed with a OneScript Plus cDNA Synthesis Kit (Abm), and cDNAs were amplified in qPCR experiments with 2× SensiFAST SYBR Lo-ROX Mix (Bioline) following the manufacturer’s instructions. The primer sequences used for gene amplification were as follows: for NRF2, the forward primer was 5′-CACATCCAGACAGACACCAGT and the reverse primer was 5′-GGGAATGTCTCTGCCAAAAGC; for GPX4, the forward primer was 5′-GGACGAGGGGAGGAGC and the reverse primer was 5′-ACGCGCACATGGTCCC; for NQO1, the forward primer was 5′-AGTTTGCTTACACTTACGCTGC and the reverse primer was 5′-CCCTTGCAGAGAGTACATGGAG; for SOD1, the forward primer was 5′-GGTGTGGCCGATGTGTCTAT and the reverse primer was 5′-CCTTTGCCCAAGTCATCTGC; for SOD2, the forward primer was 5′-GTTGGGGTTGGCTTGGTTTC and the reverse primer was 5′-TGCTCCCACACATCAATCCC; and for RPL34, the forward primer was 5′-CCAGCGTTTGACATACCGAC and the reverse primer was 5′-TGCTTTCCCAACCTTCTTGGT. Target gene expression was normalized to Ribosomal protein L34 (RPL34) and to the control group and reported as “Relative expression (RPL34; vs control).”

### Immunocytochemistry

For immunocytochemistry, the cells were seeded onto glass cover slips of 10 mm diameter at a density of 4.16 × 10^4^ cells per cm^2^. After the respective treatment, cells were washed with 1× PBS to remove the excess of the culture medium, followed by the fixation of cells in 4% formaldehyde (FA) (Thermo Scientific) at room temperature for 15 min. After discarding the FA and washing the cells with 1× PBS, 0.1% Triton X-100 was added to the cells for permeabilization. On permeabilization, the fixed cells were washed again with 1× PBS, and the non-specific binding sites were blocked by incubating the cells with 5% normal goat serum (NGS) (Sigma-Aldrich) (also with 0.1% Triton X-100 and 0.05% sodium azide to prevent bacterial growth) in 1× PBS for 1 h in a humid chamber. Following the blocking step, the cells were washed with 1× PBS and incubated with the primary antibody, GAP-43 (1:100 in 5% NGS) overnight in a humid chamber at 4 °C. After removing the primary antibodies and washing the cells with 1× PBS, the cells were incubated with the corresponding secondary antibodies, conjugated with fluorophores (1:500 in 0.5% NGS) at room temperature for 1 h. After removing the secondary antibodies, the fixed cells were washed with 1× PBS and were finally mounted (1% DAPI in the mounting solution) (Invitrogen). The cells were then visualized under fluorescent microscopy, and the images were captured using ApoTome.2 microscopy (Zeiss) with a 63×/1.40 oil objective and Zeiss ZEN 3.6 (blue edition) software. Z-stack series consisted of 0.25-µm slice intervals.

### NMR Analysis

A combined extraction of both polar and lipophilic metabolites from cell tissues was performed by utilizing a methanol/chloroform/water solvents mixture as described by Beckonert and colleagues [21]. Eighty milligrams of cell pellet was weighed in a falcon tube and 320 µL of methanol and 68 µL of distilled water were added into the tube, followed by vortex homogenization. In total, 160 µL of chloroform and 160 µL of distilled water were then added to the mixture, followed by vortexing again. The samples were then placed in the fridge for 15 min at 4 °C and later centrifuged at 1000 g for 15 min at 4 °C. The upper methanol/water phase (with polar metabolites), well separated by protein and cellular debris, was collected and dried under nitrogen flow. In order to prepare the cell tissue samples for NMR analysis, the dried hydroalcoholic phase was solubilized in 750 µL of 100 mM phosphate buffer solution with 0.2 mM trimethylsilylpropanoic acid (TSP) and 2 mM sodium azide (NaN_3_) as demonstrated by Petrella and colleagues [22]. NMR analysis was carried out on a Jeol JNM-ECZ 600R (JEOL Ltd., Tokyo, Japan) operating at a proton frequency of 600.17 MHz and equipped with a Jeol 5 mm FG/RO DIGITAL AUTOTUNE probe. ^1^H NMR experiments were carried out using a Carr Purcell-Meiboom-Gill (CPMG) filter to reduce broad signals from proteins and lipids using the following parameters: 298 K, 256 scans, residual water signal suppression with a presaturation pulse, 2.0 s relaxation delay, 90 pulse of 8.3 µs, 32 K data points, 16 dummy scan, autogain, spin echo delay time of 0.400 ms, total echo time of 64 ms, loop number 160, and 9000-Hz spectral width. ^1^H spectra were then referenced to methyl group signals of TSP (*δ* *H* = 0.00 ppm) in D_2_O. The signal identification was based on literature data [15, 22, 23], standard addition, and 1H-1H TOtal Correlated SpectroscopY (TOCSY) experiment, a two-dimensional (2D) NMR technique, performed under experimental conditions previously reported [24]. Spectrum processing and signal integration were then carried out with JEOL Delta software (v5.3.1). The three biological replicates were extracted, and each was analyzed once by NMR. Relative metabolite quantification was performed by normalizing each metabolite amount to the sum of all metabolite amounts in each spectrum (total metabolite sum), and the resulting dataset was autoscaled prior to statistical analyses [25].

### MitoTracker Staining and Mitochondrial Analysis

For MitoTracker staining procedure, the cells were seeded onto glass cover slips of 10 mm diameter at a density of 4.16 × 10^4^ cells per cm^2^. MitoTracker is a far red-fluorescent dye that stains the mitochondria by binding to the thiol-reactive chloromethyl groups in the mitochondrial membrane of live cells and its accumulation is dependent upon the mitochondrial membrane potential. This dye can be retained even after cell fixation, thereby making it possible to evaluate the different parameters regarding mitochondrial biology in live cells such as the shape of the mitochondria (large/round, rod, punctate and networks), mitochondrial footprint, branch length mean, and network branches mean [26]. The lyophilized MitoTracker (Invitrogen) was dissolved in dimethyl sulfoxide (Fisher Bioreagents) to prepare a 1-mM stock solution, which was then stored at −20 °C and away from light. This stock was then diluted to a working concentration of 10 µM by dissolving in the serum-free media, followed by directly adding it into the media (into each of the wells), such that a final concentration of 100 nM was achieved. The cells were then incubated with MitoTracker for 10 min without the presence of light. The media with the dye was aspirated, and the cells were washed with 1× PBS which was warmed to 37 °C, followed by the fixation of cells in 4% FA at room temperature for 15 min. After discarding the FA and washing the cells with 1× PBS, 0.1% Triton X-100 was added to the cells for permeabilization. On permeabilization, the fixed cells were washed again with 1× PBS and were finally mounted (1% DAPI in the mounting solution). The cells were then visualized under fluorescent ApoTome.2 microscopy (Zeiss) with a 63×/1.40 oil objective, and the images were captured using Zeiss ZEN 3.6 (blue edition) software. The red fluorescence from the MitoTracker was detected using a 540–552-nm excitation and 590–660-nm emission filter set. Z-stack series consisted of 0.25-µm slice intervals. Three independent biological replicates were performed. For each biological replicate, two technical replicates (two coverslips) were analyzed. From each coverslip, three non-overlapping fields were acquired, and three cells were randomly selected per field for quantification. The mitochondrial analysis was then performed using the plugin Mitochondrial Network Analysis (MiNA) of the ImageJ software [27].

### EDS

RA-differentiated SH-SY5Y cells were seeded on silicon-supported substrates suitable for EDS analysis. RA-differentiated SH-SY5Y cells were washed twice with PBS and incubated for 24 h in MEM 1% FBS or MEM 1% FBS supplemented with VitB12 (0.01 µM or 1 µM). Following treatment, cells were washed with PBS and fixed with 4% formaldehyde for 15 min at room temperature. Samples were then washed with PBS and finally rinsed with distilled water. Samples were analyzed using the Zeiss Auriga Scanning Electron Microscope (Zeiss, DE) equipped with the STEM module to detect intracellular cobalt (Co) as a proxy for intracellular cobalamin. Elemental maps were acquired and Co was quantified as cps/eV and as a relative atomic fraction (at.%) among detected elements (C, N, O, Na, Si). EDS analysis on the cells was performed using a very low accelerating voltage (2.5 keV) to minimize the contribution from the silicon substrate and maximize the X-ray signal originating from the cells, while still allowing detection of the Co Lα1 line (~ 0.77 keV).

### DCFDA Staining and Cytosolic ROS Image Analysis

DCFDA staining was performed according to the manufacturer’s instructions. Briefly, the cells were seeded onto glass cover slips of 10 mm diameter at a density of 4.16 × 10^4^ cells per cm^2^ and subjected to the recovery protocol as described above. Cytosolic oxidant-dependent signal during early recovery (2 h) was assessed using 2′,7′-dichlorodihydrofluorescein diacetate (DCFDA; Sigma). DCFDA was dissolved in DMSO to prepare a 10-mM stock solution, which was stored at − 20 °C and protected from light. The stock was diluted in PBS and added to the cells to reach a final concentration of 10 µM. Cells were incubated with DCFDA for 15 min at 37 °C in the dark. After incubation, the dye-containing PBS was aspirated, and cells were washed with PBS (37 °C) three times and immediately observed. Fluorescence images were acquired at 200× magnification using a Nikon Eclipse TE300 microscope under identical acquisition settings across conditions. DCFDA fluorescence was quantified in Fiji/ImageJ using an automated workflow based on global background subtraction (rolling-ball), cell-center detection by Gaussian blurring followed by Find Maxima, and measurement of the median fluorescence intensity within a fixed circular region of interest centered on each detected cell. The percentage of DCFDA-positive cells was computed using an internal, experiment-specific threshold derived from the H_2_O_2_ condition and applied consistently to all conditions within the same replicate.

### JC-1 Staining and Mitochondrial Membrane Potential Analysis

JC-1 staining was performed according to the manufacturer’s instructions. Briefly, the cells were seeded onto glass cover slips of 10 mm diameter at a density of 4.16 × 10^4^ cells per cm^2^ and subjected to the recovery protocol as described above. Mitochondrial membrane potential (ΔΨm) during early recovery (2 h) was assessed using the JC-1 dye (Cayman Chemical). JC-1 was dissolved in DMSO to prepare a 2 mM stock solution, stored at − 20 °C and protected from light, and diluted in PBS to obtain a final concentration of 2 µM. Cells were incubated with JC-1 for 15 min at 37 °C in the dark. The dye-containing PBS was then aspirated, and cells were washed with PBS (37 °C) three times and immediately observed. Images were acquired at 200× magnification using a Nikon Eclipse TE300 microscope with identical acquisition settings across conditions for both green and red channels. Images were analyzed in Fiji/ImageJ by measuring the median fluorescence intensity in the green and red channels within a fixed region of interest per cell. Mitochondrial membrane potential was reported as the red/green fluorescence ratio (R/G).

### Raman Analysis

Raman analysis of SH-SY5Y cells was performed using the confocal inVia™ Raman spectrometer (Renishaw, UK) with a 100X short-distance objective and an excitation line at 532.1 nm. Spectral acquisition was obtained with an energy of 50 mW, an exposure time of 10 s and 3 accumulations for every cell on the spectral range of 1000–3100 cm^−1^ and in the spectral region 1000–1800 cm^−1^, several peaks were identified. For each Raman map, at least 1000 individual points were acquired for 1-s exposure time and 2 accumulations [28]. Formaldehyde-fixed cells on coverslips were subjected to single-cell Raman spectroscopy. The Raman data were processed with several steps to ensure the accuracy and reliability of the results. Initially, the background signal from the glass slide was subtracted to remove any interference from the non-cellular components. Following this, all spectra were normalized to the highest peak intensity to correct for any intensity variations between the samples. This normalization process allows for better comparison across different samples and experimental conditions.

The Raman spectra were then subjected to advanced smoothing using the Savitzky-Golay method, with an interval of 10 and a polynomial order of 3. This method effectively reduces noise while preserving the spectral features, especially the peaks of interest. Additionally, spike removal was applied, using a backward spike removal algorithm with a maximum pixel width of 5 to correct any isolated spikes that could distort the data. Finally, each spectrum was interpolated and resampled to ensure uniformity in data points, facilitating accurate peak identification.

For each treatment group, the spectra were averaged, and the Raman shift was determined using the following parameters: a peak finding threshold of 2.5% of the visible spectrum’s ordinate, a search interval of 15, and a position tolerance of 0.40% (analysis conducted using Spectragryph software). The peak values derived from the averaged spectrum of each treatment were subsequently used to extract the full width at half maximum (FWHM) values (peak intensities) across all samples. The peaks were then aligned by rounding to the nearest integer. These values were used for further analysis.

### Oil Red O (ORO) Staining

For ORO staining procedure, the cells were seeded onto glass cover slips of 10 mm diameter at a density of 4.16 × 10^4^ cells per cm^2^. ORO is a fat-soluble dye used for staining neutral lipids, namely triglycerides and cholesterol esters, in biological samples that allow the visualization of LDs [29]. Firstly, the 0.5% ORO solution was prepared by dissolving ORO powder (Sigma-Aldrich) in 60% isopropanol (Merck & Co.) and was incubated for 24 h at room temperature, followed by filtering using a 0.22 µm filter (GVS Filter Technologies), the next day. The cells were first washed with 1× PBS for 5 min each to remove the media and were then fixed with 4% FA for 20 min. After fixation, the cells were washed thrice with 1× PBS for 10, 5, and 5 min, respectively. Prior to rinsing the cells, the 0.3% ORO solution was prepared by diluting the 0.5% ORO solution with distilled water, followed by incubating the 0.3% ORO solution for 10 min at room temperature, followed by filtering again using a 0.22-µm filter. The cells were then rinsed with freshly prepared 60% isopropanol, followed by incubating them with 250 μL of 0.3% ORO for 40 min. After the incubation, the ORO solution was aspirated completely, and the cells were rinsed with 60% isopropanol again. Finally, the cells were washed thrice with 1× PBS for 10, 5, and 5 min, respectively, followed by mounting the coverslips with 1% DAPI in the mounting solution.

## Results and Discussion

### Cell Model

**Fig. 1:**
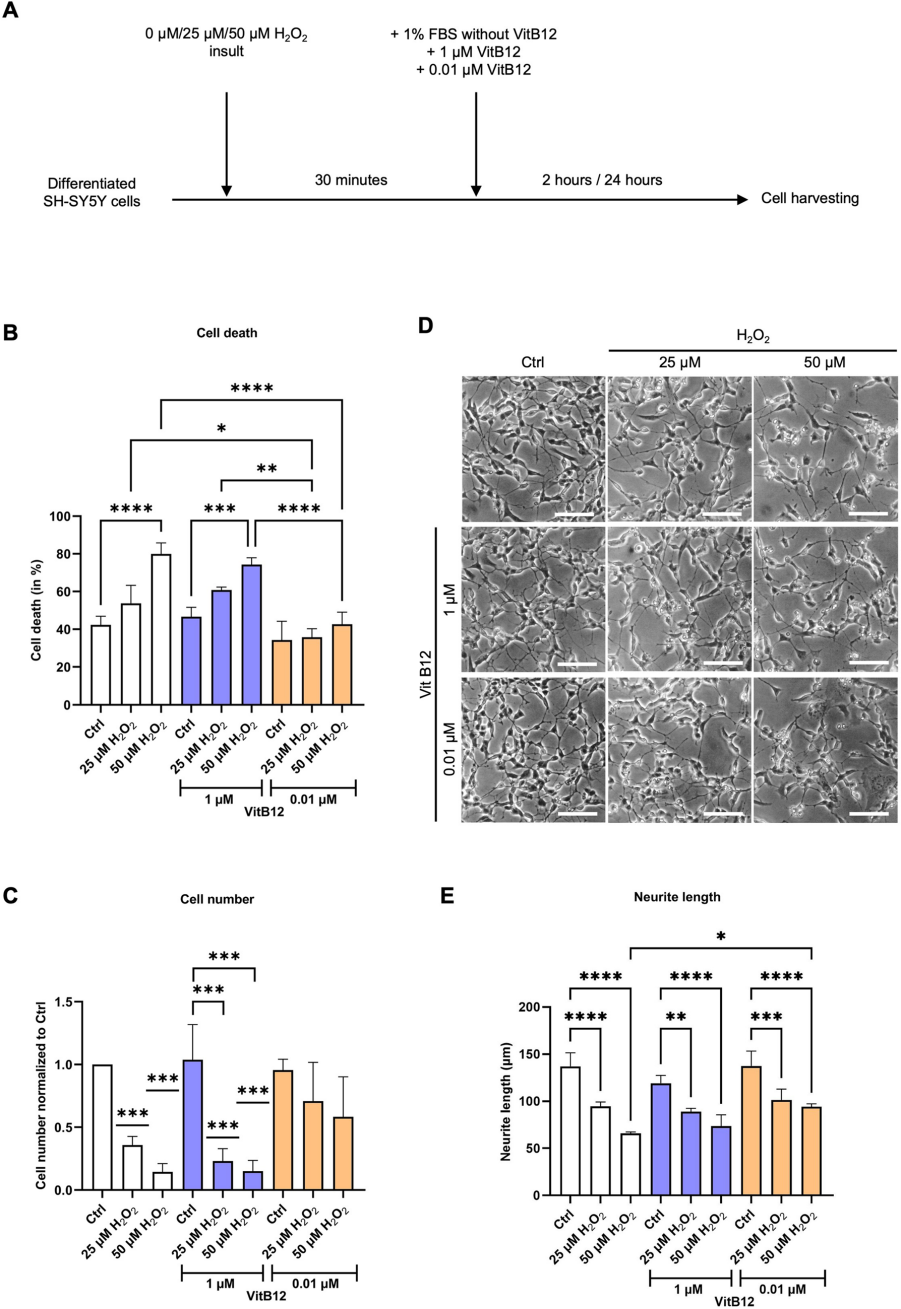
The physiological dose of vitamin B12 during recovery reduces erythrosin B–positive stained cell numbers and enhances neurite length. **A** A schematic diagram showing the experimental design including the treatments and recovery. **B** Plot for cell death in percentage for varying concentrations of H_2_O_2_ and VitB12. The conditions correspond to: Sample 1, differentiated SH-SY5Y cells in MEM containing 1% FBS; Sample 2, differentiated SH-SY5Y cells treated with 25 μM H_2_O_2_, followed by recovery in MEM containing 1% FBS; Sample 3, differentiated SH-SY5Y cells treated with 50 μM H_2_O_2_, followed by recovery in MEM containing 1% FBS; Sample 4, differentiated SH-SY5Y cells in MEM containing 1% FBS and 1 μM VitB12; Sample 5, differentiated SH-SY5Y cells treated with 25 μM H_2_O_2_, followed by recovery in MEM containing 1% FBS and 1 μM VitB12; Sample 6, differentiated SH-SY5Y cells treated with 50 μM H_2_O_2_, followed by recovery in MEM containing 1% FBS and 1 μM VitB12; Sample 7, differentiated SH-SY5Y cells in MEM containing 1% FBS and 0.01 μM VitB12; Sample 8, differentiated SH-SY5Y cells treated with 25 μM H_2_O_2_, followed by recovery in MEM containing 1% FBS and 0.01 μM VitB12; Sample 9, differentiated SH-SY5Y cells treated with 50 μM H_2_O_2_, followed by recovery in MEM containing 1% FBS and 0.01 μM VitB12. **C** Plot for cell number normalized to control (Sample 1) for varying concentrations of H_2_O_2_ and VitB12. The conditions correspond to the same as above. **D** Microscopic images of differentiated SH-SY5Y cells treated with 25 µM and 50 µM concentrations of H_2_O_2_, followed by recovery in unsupplemented and supplemented media with VitB12. Scale bar corresponds to a length of 50 µm. **E** The quantification of the neurite outgrowth processes (in µm) obtained for differentiated SH-SY5Y cells treated with 0 µM, 25 µM, and 50 µM concentration of H_2_O_2_, followed by recovery in unsupplemented and supplemented media with VitB12. The conditions correspond to the same as above. Data are from 9 biological replicates and presented as mean ± SD. Statistical significance was determined using one-way ANOVA test, followed by Tukey’s test which compared the means of two or more independent groups: *p < 0.05, **p < 0.01, ***p < 0.001, ****p < 0.0001. For cell number, comparison with Sample 1 was performed by using one sample *t*-test

SH-SY5Y cells are widely used as an in vitro neural model to study neurodevelopment and regeneration, following traumatic injury [30, 31]. Differentiation is a crucial process required to generate an SH-SY5Y cell population that exhibits morphological and functional characteristics similar to those of neurons [31]. In order to employ an in vitro neurite outgrowth assay for assessing the role of VitB12 on a neuron-like model during recovery, after genotoxic stress imposed by H_2_O_2_, we optimized a cellular system at first (Supplementary, Fig. 1S). As shown in Fig. 1S, a 3-day RA protocol under our conditions reduces the proliferative marker PCNA and increases neuronal markers (e.g., SYP) and induces clear neurite outgrowth. Moreover, neurite length reaches a plateau at later differentiation times (6–9 days; Fig. 1S), supporting our decision to use the 3-day window to preserve a measurable dynamic range in which treatment-dependent pro-neuronal versus anti-neuronal effects during recovery can still be detected.

### Experimental Model

**Fig. 2:**
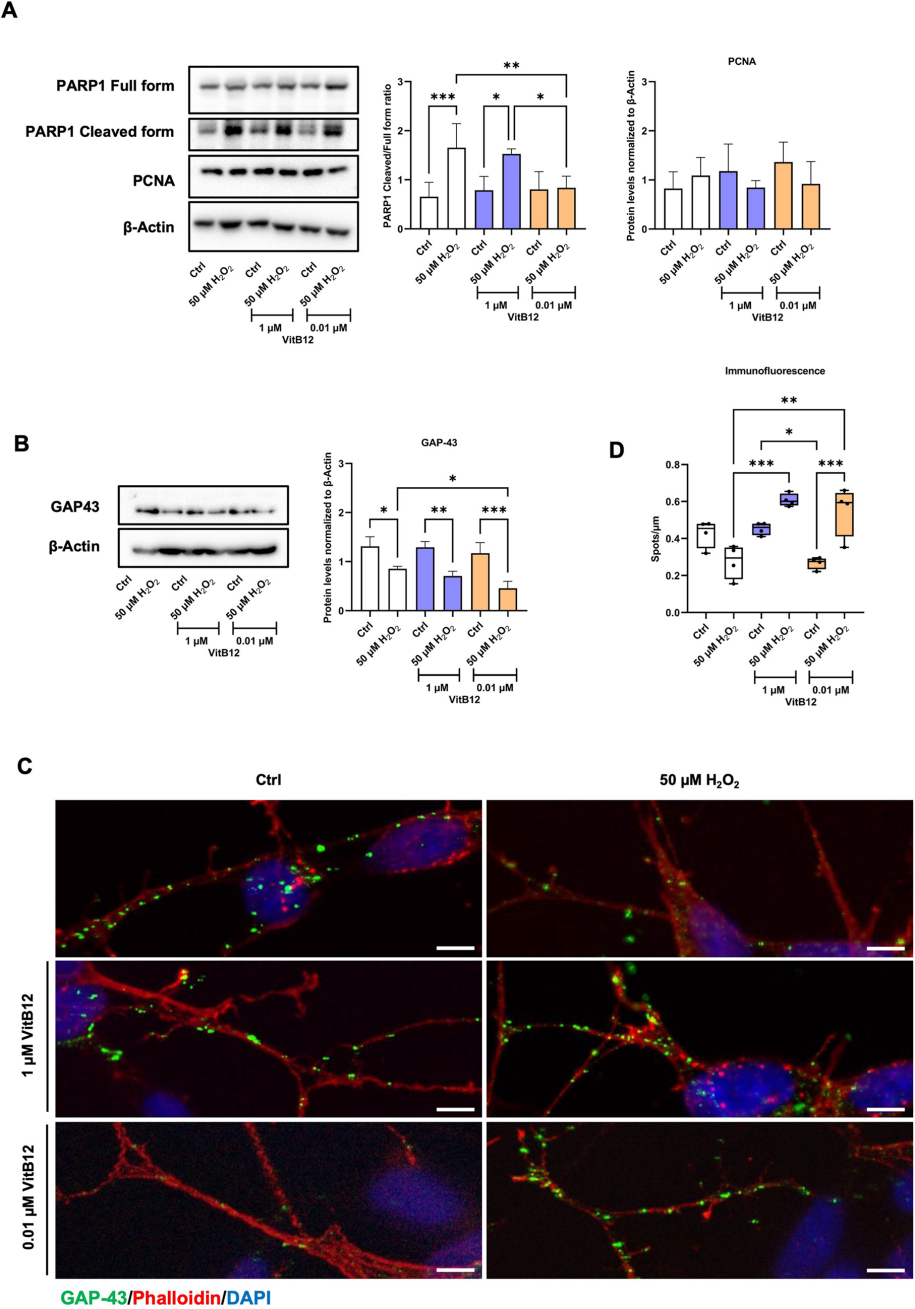
The physiological dose of vitamin B12 enhances cell survival and neurite elongation during recovery. **A** The western blots and densitometry quantification of PARP1 full form, PARP1 cleaved form, PCNA and β-Actin. β-Actin was employed as the internal reference protein. The samples correspond to: Lane 1, differentiated SH-SY5Y cells in MEM containing 1% FBS; Lane 2, differentiated SH-SY5Y cells treated with 50 μM H_2_O_2_, followed by recovery in MEM containing 1% FBS; Lane 3, differentiated SH-SY5Y cells in MEM containing 1% FBS and 1 μM VitB12; Lane 4, differentiated SH-SY5Y cells treated with 50 μM H_2_O_2_, followed by recovery in MEM containing 1% FBS and 1 μM VitB12; Lane 5, differentiated SH-SY5Y cells in MEM containing 1% FBS and 0.01 μM VitB12; Lane 6, differentiated SH-SY5Y cells treated with 50 μM H_2_O_2_, followed by recovery in MEM containing 1% FBS and 0.01 μM VitB12. The densitometric analysis was performed by evaluating the protein expression levels from the protein bands. For PARP1, the ratio of PARP1 cleaved to full form was quantified, and for PCNA, the protein expression levels were evaluated from the protein bands, followed by normalization with the bands of β-Actin. **B** The western blots and densitometry quantification of GAP-43 and β-Actin. The densitometric analysis was performed by evaluating the protein expression levels from the protein bands, followed by normalization with the bands of β-Actin. **C** Immunofluorescence microscopic images of GAP-43 at 24 h for the same conditions as that of the western blot, and the scale bar corresponds to 20 µm. **D** Immunofluorescence analysis of GAP-43 at 24 h where the aggregates of GAP-43 were measured as spot per µm of the neurite length. Data are from 3 biological replicates for the WB analysis and from 4 biological replicates for the immunofluorescence analysis, and is presented as mean ± SD. Statistical significance was determined using one-way ANOVA test, followed by Tukey’s test which compared the means of two or more independent groups: *p < 0.05, **p < 0.01, ***p < 0.001

To assess the potential beneficial effects of VitB12 in aiding recovery after H_2_O_2_ insult, we subjected RA-differentiated SH-SY5Y cells to MEM (non-insulted cells) or two different concentrations of H_2_O_2_ (25 µM and 50 µM) for 30 min in MEM. Subsequently, we allowed the cells to recover in unsupplemented medium (MEM + 1% FBS) or media supplemented with VitB12 at concentrations of 0.01 µM (physiological dose) or 1 µM (high dose) for a duration of either 2 h or 24 h. Here, the term “physiological dose” refers to a low-nanomolar supplementation in the tissue/brain-relevant order of magnitude, rather than a serum-mimicking concentration. Human post-mortem brain cobalamin content has been reported at 50–60-ng/g wet tissue, corresponding to ~ 36.9–44.3 pmol/g (i.e., ~ 36.9–44.3 nM-equivalent, assuming 1 g ≈ 1 mL) [32]. Consistently, rodent brain VitB12 measurements are reported in the pg/mg (ng/g) wet-weight range; in the TCblR/CD320 knockout mouse study, median brain VitB12 concentrations were 112.42 pg/mg in controls versus 8.59 pg/mg in KO animals, corresponding to ~ 83.0 pmol/g (~ 83.0 nM-equivalent) versus ~ 6.34 pmol/g (~ 6.34 nM-equivalent), respectively [33]. We do not imply direct equivalence to brain pharmacokinetics or CSF exposure; the term is used to denote a low-dose, tissue-plausible regime to probe dose-dependent recovery responses in vitro. To support intracellular entry under our conditions, we performed EDS mapping of intracellular cobalt as a proxy for intracellular VitB12 [34]. Because intracellular cobalt is expected to be extremely low, the EDS spectrum shows a small Co Lα1 peak in the VitB12-treated sample (near the instrumental detection limit), whereas this peak is absent in unsupplemented cells. This supports the conclusion that VitB12-derived cobalt becomes detectable intracellularly upon vitamin treatment (Fig. 2S, Table 1S). Note that the unsupplemented media does not contain any traces of VitB12, thus establishing this “basal condition” as VitB12 deprivation condition [15] (Experimental design, Fig. 1A). Recovery duration was selected based on the expected temporal dynamics of the biological response. Specifically, we included an early recovery time point (2 h) to capture rapid stress-responsive transcriptional signaling and early functional redox/mitochondrial readouts, and a later time point (24 h) to quantify more stable recovery-related phenotypes that require time to develop, such as changes in cell survival, neurite-associated structural remodeling, mitochondrial network organization, and metabolic/lipid reprogramming. Accordingly, gene-expression analyses were performed at both 2 h and 24 h, whereas all other endpoints were assessed at 24 h unless otherwise stated.

### Recovery with a Physiological Dose of VitB12 Reduces the Number of Erythrosin B–Stained Positive Cells and Enhances Neurite Length

The H_2_O_2_ insult induced a dose-dependent positive erythrosin B staining of the cells, suggesting an increase in cell death, irrespective of whether the cells were allowed to recover under unsupplemented conditions or with a high dose of VitB12 (1 µM) (Fig. 1B). Correspondingly, the cell number was reduced under these conditions (Fig. 1C). Interestingly, when cells recovered in the media supplemented with a physiological dose of VitB12 (0.01 µM VitB12), the impact of H_2_O_2_ exposure on cell death was diminished (Fig. 1B), which coincided with a higher number of cells compared to the other conditions (Fig. 1C). No differences in the mentioned parameters were observed between the supplemented conditions and the unsupplemented condition in the absence of H_2_O_2_ insult (Fig. 1B, C). 

Regarding the neurite outgrowth, supplementation with the physiological or high doses of VitB12 did not alter the neurite length, compared to the unsupplemented medium (Fig. 1D, E). Furthermore, a significant decrease in neurite length was observed for the cells treated with H_2_O_2_ both at 25 µM and 50 µM, irrespective of the recovery media type. Interestingly, on comparing the neurites of cells treated with 50 µM H_2_O_2_ and recovering in unsupplemented media versus those recovering in the physiological concentration of VitB12, the latter condition resulted in longer neurites, suggesting a potential beneficial effect. In contrast, a high dose of VitB12 did not improve recovery after H_2_O_2_ insult, and the effects were comparable to that of the unsupplemented media (Fig. 1D, E). Notably, the physiological VitB12 condition shows increased neurite length despite higher cell survival and cell number (Fig. 1C). This argues against a sparse-culture artifact due to H_2_O_2_-induced cell loss and suggests that the neurite-elongation effect is not driven by reduced confluence.

Since our system demonstrated substantial differences only after the insult with 50 µM H_2_O_2_, we focused on this concentration and did not further investigate the effects of the lower H_2_O_2_ dose.

### Physiological Levels of Vitamin B12 Enhance Cell Survival and Neurite Elongation

**Fig. 3:**
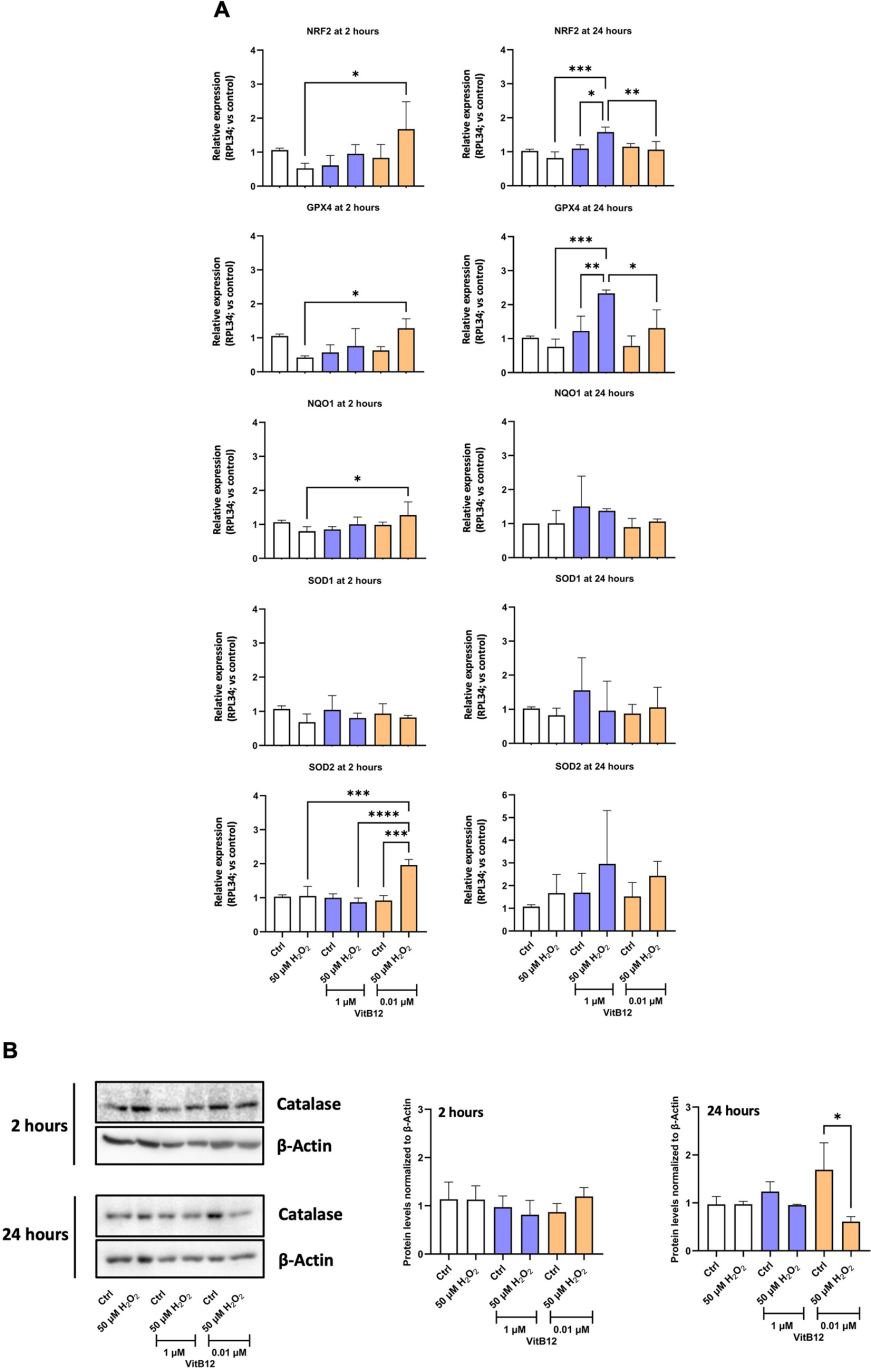
The physiological dose of vitamin B12 activates antioxidative stress defenses earlier than the high dose in H_2_O_2_-insulted SH-SY5Y cells. **A** The qRT-PCR gene expression levels of NRF2, GPX4, NQO1, SOD1, and SOD2, at 2 h and 24 h, with respect to the housekeeping gene RPL34. The samples are: Condition 1, differentiated SH-SY5Y cells in MEM containing 1% FBS; Condition 2, differentiated SH-SY5Y cells treated with 50 μM H_2_O_2_, followed by recovery in MEM containing 1% FBS; Condition 3, differentiated SH-SY5Y cells in MEM containing 1% FBS and 1 μM VitB12; Condition 4, differentiated SH-SY5Y cells treated with 50 μM H_2_O_2_, followed by recovery in MEM containing 1% FBS and 1 μM VitB12; Condition 5, differentiated SH-SY5Y cells in MEM containing 1% FBS and 0.01 μM VitB12; Condition 6, differentiated SH-SY5Y cells treated with 50 μM H_2_O_2_, followed by recovery in MEM containing 1% FBS and 0.01 μM VitB12. **B** The western blots of catalase and β-actin, with their corresponding densitometric analysis. The densitometric analysis was performed by evaluating the protein expression levels from the protein bands, followed by normalization with the bands of β-Actin. Data are from 3 biological replicates and are presented as mean ± SD. Statistical significance was determined using one-way ANOVA test, followed by Tukey’s test which compared the means of two or more independent groups: *p < 0.05, **p < 0.01, ***p < 0.001

To corroborate the differences in cell death observed as a result of erythrosin B staining, we evaluated the expression levels of PARP1 (directly associated with DNA damage) [35], by WB (Fig. 2A). Treatment with 50 µM H_2_O_2_, followed by recovery in either an unsupplemented medium or a high dose of VitB12, resulted in an increased relative abundance of the cleaved (inactive) form of PARP1. In contrast, cells recovering with the physiological levels of VitB12 exhibited a lower relative proportion of cleaved PARP1, indicating a potential protective effect of the treatment. This suggests that cell recovery in the physiological dose of VitB12 after exposure to 50 µM H_2_O_2_ results in reduced apoptosis. This is in accord with the reduced caspase-3 activation, with PARP1 being one of its targets [35] (Fig. 3S). Additionally, we measured the PCNA levels, which remained unchanged across all conditions, suggesting the absence of active cell proliferation (Fig. 2A). Overall, the increased cell number observed during recovery with the physiological doses of VitB12 (Fig. 1C) is a result of reduced cell death.

To corroborate the morphological data on neurite elongation, we biochemically measured the cellular levels of GAP-43 (Fig. 2B). The cells cultured only in unsupplemented, high-dose, or physiological dose of VitB12 conditions did not result in significant differences. Cells exposed to 50 µM H_2_O_2_ showed a general reduction of GAP-43, but the ones recovering in unsupplemented medium or under high-dose VitB12 conditions showed higher GAP-43 levels compared to the cells recovering in the physiological dose. To further investigate this, we immunolocalized GAP-43 on neurites and quantified the number of dots relative to neurite length (Fig. 2C, D). Non-H_2_O_2_-insulted cells exhibit a similar number of GAP-43 dots per micrometer of neurite length. Conversely, after treatment with 50 µM H_2_O_2_, the recovery medium produced distinct outcomes: recovery in an unsupplemented medium did not significantly change the number of dots (although a decreasing trend is observable compared to its non-insulted counterpart), whereas both the physiological and high-dose treatments led to an increased number of dots (Fig. 2C, D). The lack of correlation between the number of GAP-43 dots in neurites and the total protein levels detected by WB analysis could be attributed to differences in the subcellular localization of GAP-43 (distribution between the cell body and neurites). Thus, while the physiological dose of VitB12 induces a global reduction in GAP-43 protein levels, the concomitant increase in the number of dots per micrometer in neurites and the greater neurite elongation observed from morphological assessments (Fig. 1E) suggests that the physiological doses of VitB12 effectively promote neurite elongation. The fact that recovery under high-dose conditions results in a comparable number of GAP-43 dots per micrometer of neurite length as the physiological dose, but with a greater amount of GAP-43 protein level, suggests that the cells recovering under high-dose conditions may still be in an early phase of GAP-43 accumulation before neurite elongation occurs.

### The Physiological Dose of Vitamin B12 Triggers an Early Increase in the Expression of Antioxidant Stress-Associated Genes in H_2_O_2_-Insulted SH-SY5Y Cells

**Fig. 4:**
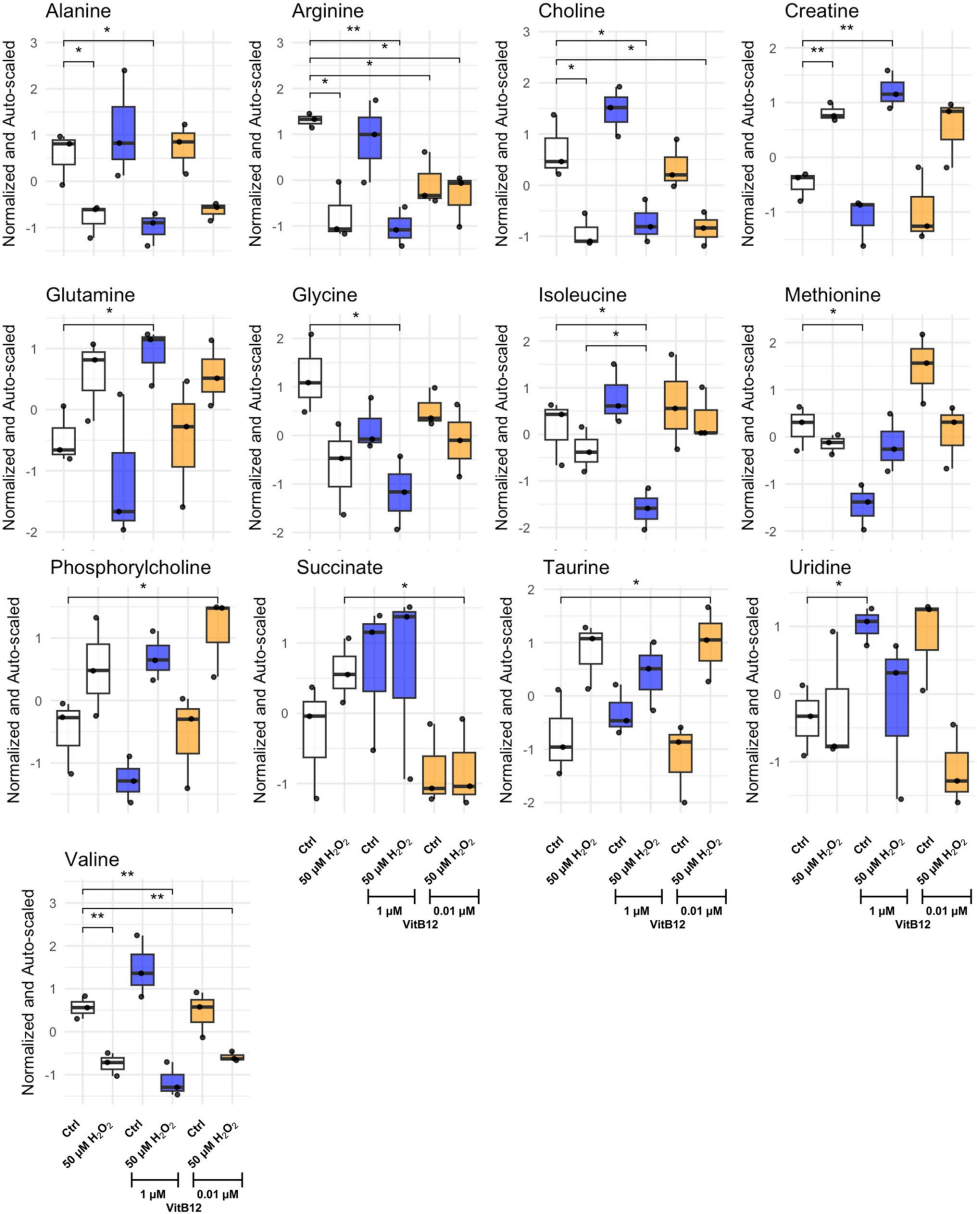
The physiological dose of vitamin B12 decreases succinate levels in H_2_O_2_-insulted SH-SY5Y cells. The metabolic profile alterations (differences in the metabolites) are from the endo-metabolome analysis. The conditions correspond to: Condition 1, differentiated SH-SY5Y cells in MEM containing 1% FBS; Condition 2, differentiated SH-SY5Y cells treated with 50 μM H_2_O_2_, followed by recovery in MEM containing 1% FBS; Condition 3, differentiated SH-SY5Y cells in MEM containing 1% FBS and 1 μM VitB12; Condition 4, differentiated SH-SY5Y cells treated with 50 μM H_2_O_2_, followed by recovery in MEM containing 1% FBS and 1 μM VitB12; Condition 5, differentiated SH-SY5Y cells in MEM containing 1% FBS and 0.01 μM VitB12; Condition 6, differentiated SH-SY5Y cells treated with 50 μM H_2_O_2_, followed by recovery in MEM containing 1% FBS and 0.01 μM VitB12. Data are from 3 biological replicates and are presented as mean ± SD. Metabolite abundances were normalized to the total metabolite sum (sum of all metabolites per spectrum) and auto-scaled prior to statistical analysis. Statistical significance was determined using one-way ANOVA (*p* < 0.2), followed by Tukey's test which compared the means of two or more independent groups: *p < 0.05, **p < 0.01, ***p < 0.001

**Fig. 5:**
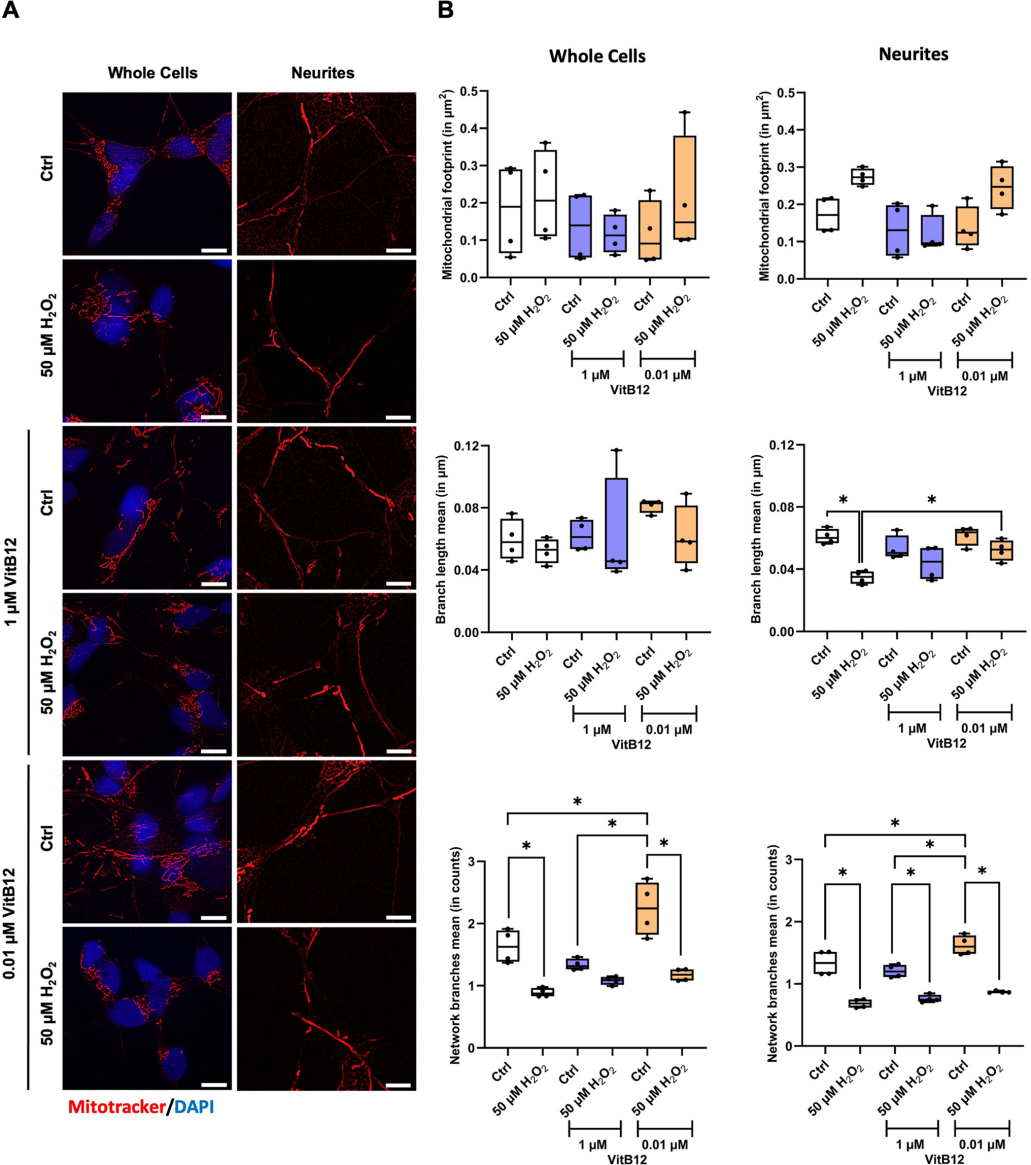
The physiological concentration of vitamin B12 enhances mitochondrial morphodynamics in H_2_O_2_insulted SH-SY5Y cells. **A** Fluorescence microscopic images of the MitoTracker-stained (the scale bar corresponds to a length of 20 µm) and **B** relative mitochondrial analysis (performed using the plugin mitochondrial network analysis, MiNA, of the ImageJ software; the plots report quantification of the mitochondrial footprint (in µm2), branch length mean (in µm), and network branches mean (as counts) parameters of the individual cells and neurites. The conditions correspond to: Condition 1, differentiated SH-SY5Y cells in MEM containing 1% FBS; Condition 2, differentiated SH-SY5Y cells treated with 50 μM H_2_O_2_, followed by recovery in MEM containing 1% FBS; Condition 3, differentiated SH-SY5Y cells in MEM containing 1% FBS and 1 μM VitB12; Condition 4, differentiated SH-SY5Y cells treated with 50 μM H_2_O_2_, followed by recovery in MEM containing 1% FBS and 1 μM VitB12; Condition 5, differentiated SH-SY5Y cells in MEM containing 1% FBS and 0.01 μM VitB12; Condition 6, differentiated SH-SY5Y cells treated with 50 μM H_2_O_2_, followed by recovery in MEM containing 1% FBS and 0.01 μM VitB12. Data are from 4 biological replicates and are presented as mean ± SD. Statistical significance was determined using one-way ANOVA test, followed by Tukey's test which compared the means of two or more independent groups: *p < 0.05

The morphological and molecular characterization of cellular survival and recovery parameters suggests a potential beneficial effect of the physiological dose of VitB12 in mitigating H_2_O_2_-induced damage. To gain deeper insight into the molecular mechanisms underlying the protective effects of VitB12, we analyzed the expression levels of key genes involved in antioxidative stress responses, including the mRNA levels of the transcription factor NF-E2 p45-related factor 2 (*NFE2L2* or *NRF2*), and that of its target genes encoding enzymes capable of shielding the cell from the adverse effects induced by oxygen radicals, including glutathione peroxidase 4 (*GPX4*), NAD(P)H:quinone oxidoreductase 1 (*NQO1*), superoxide dismutase 1 (*SOD1*), and superoxide dismutase 2 (*SOD2*) [36]. NRF2 activity is strictly regulated by imbalances in the cellular redox homeostasis, as oxidative insults determine its activation [37], thereby increasing the expression of *GPX4*, which protects cells by reducing lipid hydroperoxides, preserving membrane integrity [38]; of *NQO1*, enhancing antioxidant capacity as part of the NRF2-mediated response [39]; and of *SOD1* and *SOD2*, that convert superoxide radicals into less harmful molecules in the cytoplasm and the mitochondria, respectively [40]. Considering that mentioned genes could have different time-related dynamics for their expression, we decided to assess their expression at 2 h (early stage) and 24 h (later stage), the latter being the time frame used in most of the experiments in this study. Because early antioxidant transcriptional activation precedes morphological recovery, we interpret the 2 h gene-expression response as an early stress-response layer that may shape the consolidated recovery phenotypes quantified at 24 h (MTT viability and neurite outgrowth). To strengthen the early-phase interpretation, we complemented qRT-PCR with DCFDA and JC-1 readouts at 2 h (Figs. 4S–5S).

Overall, antioxidant markers appear to be upregulated earlier in response to a physiological dose of VitB12 than with a high dose, following H_2_O_2_ exposure. The distinct responses observed at these two concentrations are notable, as they suggest a faster recovery with the physiological dose, and a delayed response with the high dose of VitB12 (Fig. 3A). This trend was further supported by the catalase (CAT) protein analysis, an enzyme that mitigates oxidative damage by converting H_2_O_2_ into water and oxygen [41]. Considering that protein translation events are generally delayed relative to transcriptional activation [42], this analysis revealed an increase in the CAT levels only after 24 h of treatment with the physiological dose of VitB12 (Fig. 3B). To complement the early transcriptional antioxidant response (2 h), we directly assessed cytosolic redox status and mitochondrial functional resilience during recovery. Specifically, DCFDA fluorescence imaging was used to quantify the fraction of cells showing elevated oxidant-dependent signal, and JC-1 was used as a functional readout of mitochondrial membrane potential (ΔΨm) at 2 h (Figs. 4S–5S). At 2 h, DCFDA fluorescent staining showed a reduction in the fraction of cells with elevated oxidant-dependent signal during recovery with VitB12, with the physiological dose bringing values closer to controls and the high dose producing a stronger suppression of the DCFDA-positive fraction. JC-1 analysis showed an increased red/green ratio specifically with the physiological dose in H_2_O_2_-insulted cells, consistent with a more polarized mitochondrial membrane potential (ΔΨm). Together with the 2 h gene-expression response, these assays provide complementary support that VitB12 dose influences early redox dynamics and mitochondrial functional status during recovery. Notably, the marked suppression of DCFDA positivity at the high dose suggests an early redox shift, highlighting that effective recovery likely depends on restoring an appropriate redox balance rather than maximally suppressing oxidant-dependent signals.

### Physiological Dose of Vitamin B12 Reduces Succinate in H_2_O_2_-Insulted Cells

Our observations indicate differences in cell death and neurite elongation after H_2_O_2_ insult depending on the dose of VitB12 used during recovery. Considering the well-established role of VitB12 in metabolism [15], we decided to assess possible differences in the cellular metabolic response to these treatments using high-resolution NMR endo-metabolome analysis. This analysis allowed us to identify twelve amino acids (leucine, isoleucine, valine, alanine, arginine, glutamate, methionine, lysine, aspartate, tyrosine, phenylalanine, and glutamine), two organic acids (succinic acid and lactic acid), three choline derivatives (choline, phosphocholine, and glycerophosphocholine), and other compounds such as creatine, myo-inositol, taurine, and uridine (Table 3S).

**Fig. 6:**
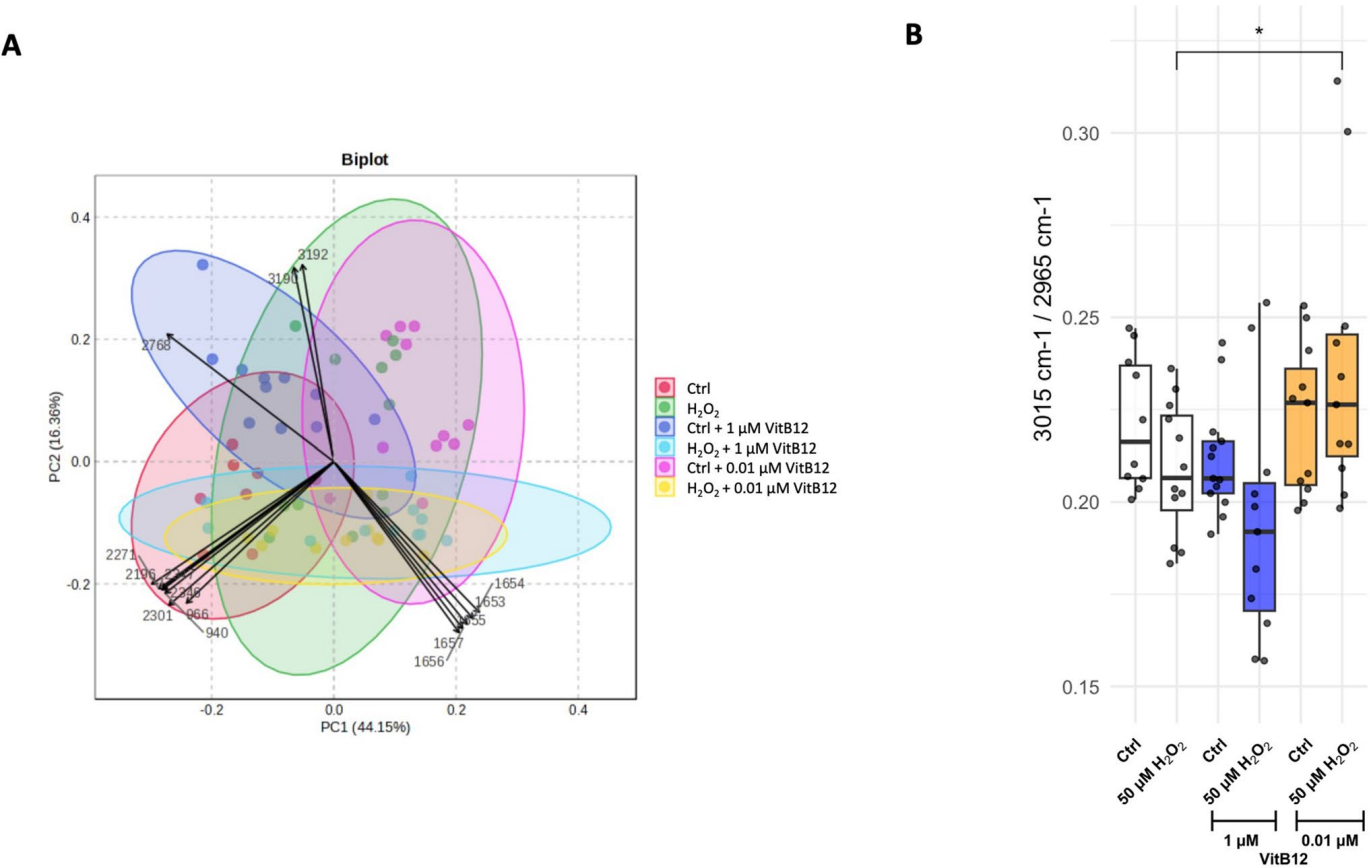
The physiological dose of vitamin B12 improves cell lipid composition in H_2_O_2_ insulted-SH-SY5Y cells as revealed by single-cell Raman spectroscopy. **A** The biplot denoting the PCA which reveals the distribution of different Raman peaks with colors indicating different experimental groups. PC1 accounts for 44.15% and PC2 accounts for 16.36% of the total variance. **B** The ratios of 2851 cm⁻^1^/2930 cm⁻^1^, 2965 cm⁻^1^/2930 cm⁻^1^, 3015 cm⁻^1^/2930 cm⁻^1^, and 3015 cm⁻^1^/2965 cm⁻^1^ calculated considering the different Raman peaks to identify the extent of lipid remodeling. The conditions correspond to: Condition 1, differentiated SH-SY5Y cells in MEM containing 1% FBS; Condition 2, differentiated SH-SY5Y cells treated with 50 μM H_2_O_2_, followed by recovery in MEM containing 1% FBS; Condition 3, differentiated SH-SY5Y cells in MEM containing 1% FBS and 1 μM VitB12; Condition 4, differentiated SH-SY5Y cells treated with 50 μM H_2_O_2_, followed by recovery in MEM containing 1% FBS and 1 μM VitB12; Condition 5, differentiated SH-SY5Y cells in MEM containing 1% FBS and 0.01 μM VitB12; Condition 6, differentiated SH-SY5Y cells treated with 50 μM H_2_O_2_, followed by recovery in MEM containing 1% FBS and 0.01 μM VitB12. Data are from 4 different coverslips and are presented as mean ± SD. Statistical significance was determined using one-way ANOVA test, followed by Tukey’s test which compared the means of two or more independent groups: *p < 0.05, **p < 0.01, ***p < 0.001, ****p < 0.0001

Multivariate analyses, i.e., principal component analysis (PCA) and pairwise PERMANOVA (Tables 1–2S and Fig. 6S A), were performed on the NMR quantitative data. Neither method revealed statistically significant differences, likely due to their focus on capturing global data structures rather than subtle variations between specific groups. However, a visual trend of sample separation in the PCA score plot was observed between H_2_O_2_-insulted cells and their non-insulted counterparts when considered as overall groups. These results prompted us for further investigation using pairwise comparisons, which could be more sensitive for detecting specific group differences. Given the exploratory nature of this metabolomics layer and the limited statistical power inherent to a multi-group (six-condition) design, we used ANOVA *p* < 0.2 as a screening threshold to flag candidate metabolites showing trend-level differences. Importantly, ANOVA *p* < 0.2 was not interpreted as confirmatory significance; specific group differences were considered supported only when passing more stringent post hoc testing (*p* < 0.05) (Fig. 4). Accordingly, pathway-level interpretation is presented as hypothesis-generating, given the limited number of quantified metabolites, and requires targeted validation. In the absence of H_2_O_2_ insult, the physiological dose of VitB12 had limited effects compared to the unsupplemented condition, with a significant decrease in arginine; in contrast, the high dose of VitB12 induced more pronounced changes, including a significant decrease in methionine and an increase in uridine. H_2_O_2_-insulted cells recovering in unsupplemented medium, compared to their non-insulted counterpart, exhibited a decrease in alanine, arginine, choline, and valine, along with an increase in creatine, reflecting widespread disruption of metabolic pathways (Fig. 4). When VitB12 was administered after H_2_O_2_-induced stress, the recovery effects varied between physiological and high doses: specifically, recovery with high-dose VitB12 supplementation resulted in a reduction in isoleucine levels without significant changes in alanine, valine, or choline; in contrast, recovery with physiological doses of VitB12 supplementation led to a decrease in succinate only. Furthermore, H_2_O_2_-insulted cells recovering with physiological levels of VitB12 showed elevated levels of phosphorylcholine and taurine compared to non-insulted cells cultured in unsupplemented medium (Fig. 4). Next, we queried MetaboAnalyst.ca to perform a pathway analysis, considering the metabolites that changed following the H_2_O_2_ treatment, and recovering in a physiological dose VitB12 supplementation. Specifically, the analysis included alanine, arginine, choline, valine, creatine, succinate, phosphorylcholine, and taurine. The pathway analysis identified the alanine, aspartate, and glutamate metabolism pathway as the most significantly affected (*p* = 0.0078265) (Fig. 6S B), which involves alanine (C00042) and succinate (C00041). Alanine and succinate play a crucial role in maintaining mitochondrial balance in the neurons, primarily through energy metabolism and the Krebs cycle. Alanine, a non-essential amino acid, is converted into pyruvate, which can either enter glycolysis or be transformed into acetyl-CoA to fuel the Krebs cycle, thereby influencing mitochondrial energy production. Similarly, succinate, a key metabolite in the Krebs cycle, can enhance ATP production by directly supplying electrons to the electron transport chain, thereby supporting mitochondrial function [43]. Overall, NMR data suggests a potential enhancement in mitochondrial activity when cells were recovered in a physiological dose of VitB12.

### VitB12 at Physiological Levels Optimizes Mitochondrial Morphodynamics

The role of VitB12 in mitochondrial activity is well-documented [44]. Interestingly, our findings suggest a potential improvement in mitochondrial function, following H_2_O_2_-induced stress and subsequent recovery only with the physiological dose of VitB12. Based on these observations, we decided to investigate potential changes in the mitochondrial morphodynamics. Mitochondria were stained using MitoTracker and the microscopic images were analyzed using the MiNA plugin of the Fiji software, which allowed us to quantify various key mitochondrial morphology parameters, including the mitochondrial footprint (the total area of the image occupied by the mitochondrial signal), the branch length mean (the average length of the lines used to represent mitochondrial structures), and the network branch mean (the average number of attached lines used to represent each structure) [26]. We evaluated these parameters both across the whole cell surface (including the cell body and neurites) and also specifically to neurites.

Based on the MiNA analysis, there was no significant variation in the mitochondrial footprint of neurites and whole cells when treated with H_2_O_2_ and allowed to recover in either unsupplemented medium or supplemented with VitB12 media (Fig. 5A, B). In terms of the mean branch length, no noticeable change was detected in the whole cells. However, on analyzing the neurites specifically, we observed a significant decrease in this parameter for the H_2_O_2_-exposed cells recovering in unsupplemented medium compared to non-exposed cells in the same medium (Fig. 5A, B). This reduction may be linked to an increase in fragmented networks resulting from mitochondrial fission (as inferred). Interestingly, when the H_2_O_2_-exposed cells were allowed to recover in a physiological dose of VitB12, the neurite branch length mean was significantly higher compared to the H_2_O_2_-exposed cells recovering in unsupplemented medium (Fig. 5A, B). This suggests a potential decrease in mitochondrial fragmentation and an overall improvement in the neurite length. Additionally, regarding the mean number of network branches, a general decline was observed following the H_2_O_2_ exposure (Fig. 5A, B). This decrease implies that mitochondrial networking was disrupted due to the H_2_O_2_ exposure, leading to mitochondrial fission and the formation of fragmented structures. Furthermore, in both the whole cells and neurites, between the group of non-H_2_O_2_-insulted cells, a physiological dose of VitB12 determined the highest level of mitochondrial networking (Fig. 5A, B). The increase in network branching may reflect enhanced mitochondrial networking and structural remodeling, consistent with improved mitochondrial organization in the presence of a physiological dose of VitB12. Overall, across the mitochondrial network parameters assessed, treatment with a physiological dose of VitB12 was associated with improved mitochondrial morphodynamics [45]. Importantly, these imaging-based observations are consistent with the direction of the exploratory NMR endo-metabolome findings (Fig. 4), providing convergent support across orthogonal approaches.

### The Recovery in a Physiological Dose of Vitamin B12 After H_2_O_2_ Insult Optimizes Lipid Composition and Promotes Formation of Lipid Droplets (LDs)

The decrease in succinate levels observed through endo-metabolome analysis, along with improvements in mitochondrial morphodynamics detected by MitoTracker, collectively indicate enhanced mitochondrial organization and activity during the recovery with a physiological dose of VitB12 after H_2_O_2_-insult. To gain deeper insight into the biochemical modifications, we conducted a single-cell Raman spectroscopic (SCRS) analysis, a powerful and label-free tool that enables biochemical profiling of individual cells without the need for external markers [28, 46–49].

**Fig. 7:**
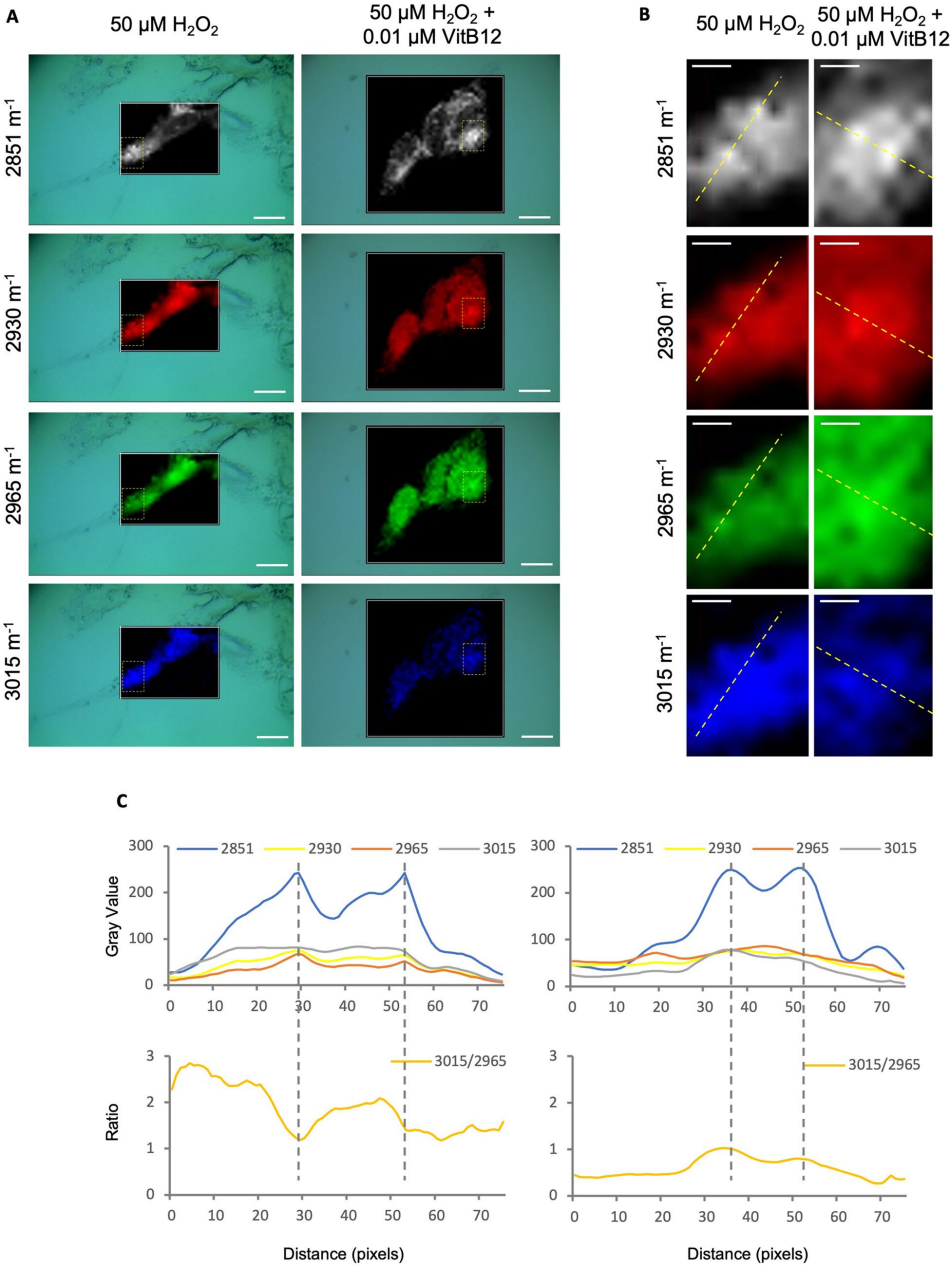
Raman mapping analysis reveals increased unsaturated lipid content in lipid droplets during recovery with physiological vitamin B12 supplementation. **A** Raman intensity map for^−^Raman shift at 2851 cm^−1^ (lipids), 2930 cm^−1^ (proteins), 2965 cm^−1^ (cholesteryl esters), and 3015 cm^−1^ (unsaturated lipids). **B** Selected segment used for profile analysis (yellow line), showing two prominent intensity peaks corresponding to LDs. **C** Intensity profiles along the selected segment for Raman shifts at 2851 cm^−1^ (lipids), 2930 cm^−1^ (proteins), 2965 cm^−1^ (cholesteryl esters), and 3015 cm^−1^ (unsaturated lipids, and 3015/2965 cm^−1^ ratio. The samples correspond to: Condition 1, differentiated SH-SY5Y cells treated with 50 μM H_2_O_2_, followed by recovery in MEM containing 1% FBS; Condition 2, differentiated SH-SY5Y cells treated with 50 μM H_2_O_2_, followed by recovery in MEM containing 1% FBS and 0.01 μM VitB12. For panel A, the scale bar indicates 10 µm, corresponding to 72 pixels; for panel B, the scale bar indicates 2.5 µm, also corresponding to 72 pixels

SCRS data (mean of Raman spectra reported in Fig. 6 and 7S), obtained by analyzing 1 μm^2^ regions of individual cell surfaces, were subjected to PCA and pairwise PERMANOVA. PCA results showed that the first two principal components accounted for a substantial proportion of the total variance, with PC1 explaining 44.15% and PC2 16.36% (Fig. 6A, Table 1). The corresponding biplot (Fig. 6A) revealed distinct spectral features contributing to this variance, notably Raman shifts at 3190 cm⁻^1^ and 3192 cm⁻^1^, strongly associated with PC1 and attributed to O–H and N–H stretching vibrations linked to water content and protein hydration [28, 46–49]. Additional discriminative features included a shift at 2768 cm⁻^1^, indicative of lipid-related CH overtones, and a cluster of shifts between 2196 and 2347 cm⁻^1^, corresponding to C≡C and C≡N bonds—suggestive of alterations in lipid composition and potential lipid peroxidation [28, 46–49]. Raman shifts within the Amide I region (1653–1657 cm⁻^1^) and in the protein backbone region (940–966 cm⁻^1^) further indicated changes in protein conformation and organization [28, 46–49]. Then, we applied pairwise PERMANOVA to quantitatively assess which group comparisons showed significant differences in their overall spectral profiles (Table 2). Significant differences were observed across all experimental comparisons, except between H_2_O_2_-insulted cells recovering with a physiological dose of VitB12 versus those recovering with a high dose. The spectral variations and statistical outcomes indicate that the experimental groups can be clearly distinguished based on global cellular chemical composition, particularly reflecting alterations in protein structure and lipid composition associated with oxidative stress and recovery. In addition, endo-metabolome analysis revealed elevated levels of phosphorylcholine and taurine in cells exposed to H_2_O_2_ and subsequently treated with a physiological dose of VitB12, compared to non-insulted cells cultured in unsupplemented medium (Fig. 4). Overall, the combined evidence from SCRS and endo-metabolome analyses suggests that physiological levels of VitB12 may contribute not only to the stabilization of membrane integrity but also to broader improvements in lipid metabolism [50–52].

**Table 1:** PCA loadings of Raman shifts from single-cell Raman spectroscopy (SCRS) analysis

Raman shift (cm⁻^1^)	PC1	PC2	Raman shift (cm⁻^1^)	PC1	PC2	Raman shift (cm⁻^1^)	PC1	PC2	Raman shift (cm⁻^1^)	PC1	PC2
211	−0.061282	0.088356	753	−0.085903	0.10886	1446	0.11588	−0.091705	2375	−0.11734	−0.11246
295	−0.12094	0.012272	778	−0.094738	0.064678	1447	0.11603	−0.089076	2427	−0.12981	−0.064982
310	−0.12314	−0.0034399	822	−0.10034	0.080785	1572	−0.019543	−0.10572	2466	−0.13307	−0.044273
338	−0.11902	−0.012586	849	0.019683	0.10504	1573	−0.020738	−0.10619	2512	−0.13509	−0.035047
371	−0.12769	−0.033029	882	−0.091368	−0.008055	1574	−0.021597	−0.10656	2550	−0.13502	−0.019108
400	−0.12344	−0.064977	940	−0.10693	−0.13685	1576	−0.018503	−0.10971	2608	−0.13008	0.021928
411	−0.11354	−0.07304	966	−0.093353	−0.14714	1653	0.092143	−0.15671	2609	−0.13157	0.019968
444	−0.11114	−0.080762	999	0.041772	−0.034468	1654	0.088185	−0.16313	2650	−0.12009	0.065013
470	−0.11895	−0.094316	1028	−0.092932	−0.086072	1655	0.08435	−0.16904	2683	−0.11745	0.084504
476	−0.12678	−0.079831	1049	−0.09836	−0.034123	1656	0.081652	−0.17371	2685	−0.11158	0.091968
501	−0.10925	−0.11171	1053	−0.096422	−0.066538	1657	0.07926	−0.17774	2728	−0.045361	0.11611
518	−0.10526	−0.053841	1094	−0.095217	−0.089041	1734	−0.072636	−0.093865	2729	−0.052822	0.12119
521	−0.09946	−0.042036	1123	−0.062166	−0.04645	1802	−0.12627	−0.047946	2730	−0.060853	0.12379
534	−0.05223	0.033734	1172	−0.11859	−0.0011121	1864	−0.1309	−0.022254	2731	−0.068274	0.12267
539	−0.059155	0.035412	1202	−0.080399	−0.050212	1921	−0.13464	−0.010929	2768	−0.10558	0.13246
542	−0.070782	0.038377	1248	−0.043288	−0.11013	2006	−0.13763	−0.034853	2929	0.1326	0.085308
546	−0.093408	0.030363	1258	−0.032924	−0.14161	2083	−0.13376	−0.050582	2930	0.13248	0.085422
576	−0.11169	0.07842	1260	−0.034554	−0.13799	2121	−0.1291	−0.072611	2931	0.13243	0.085143
596	−0.11831	0.072616	1297	0.03283	−0.11098	2137	−0.13081	−0.082755	2932	0.13211	0.085669
620	−0.10501	0.11951	1303	0.021991	−0.10997	2196	−0.11534	−0.12734	3055	−0.022008	0.12258
625	−0.12399	0.096226	1332	−0.013752	−0.077815	2271	−0.11088	−0.13221	3056	−0.018859	0.12017
640	−0.10668	0.10333	1368	−0.029685	0.0092186	2301	−0.10451	−0.14942	3057	−0.016732	0.11673
667	−0.096917	0.1231	1442	0.11345	−0.09883	2324	−0.1055	0.093328	3058	−0.015158	0.1124
679	−0.10102	0.11484	1443	0.11465	−0.096681	2346	−0.10881	−0.13343	3190	−0.025282	0.2018
722	−0.098478	0.074155	1444	0.11536	−0.094691	2347	−0.10933	−0.13244	3192	−0.019949	0.20471
747	−0.08505	0.10272	1445	0.11562	−0.093626	2372	−0.11866	−0.10904

**Table 2:** Pair-wise PERMANOVA results from single-cell Raman spectroscopy

Comparison	F.model	*R*^2^	*p*val	p.adj
0.010 µM VitB12 vs 1 µM VitB12	32.10000	0.59335	0.00100	0.00214
0.010 µM VitB12 vs Ctrl	56.30400	0.74769	0.00100	0.00214
0.010 µM VitB12 vs 50 µM H_2_O_2_	2.77480	0.21721	0.01300	0.01625
0.010 µM VitB12 vs 50 µM H_2_O_2_ + 0.010 µM VitB12	11.85400	0.37213	0.00100	0.00214
0.010 µM VitB12 vs 50 µM H_2_O_2_ + 1 µM VitB12	5.19350	0.20614	0.01300	0.01625
1 µM VitB12 vs Ctrl	9.25320	0.30586	0.00300	0.00500
1 µM VitB12 vs 50 µM H_2_O_2_	11.80100	0.33910	0.00100	0.00214
1 µM VitB12 vs 50 µM H_2_O_2_ + 0.010 µM VitB12	12.22000	0.35711	0.00100	0.00214
1 µM VitB12 vs 50 µM H_2_O_2_ + 1 µM VitB12	8.73890	0.45423	0.00100	0.00214
Ctrl vs 50 µM H_2_O_2_	22.45100	0.52887	0.00100	0.00214
Ctrl vs 50 µM H_2_O_2_ + 0.010 µM VitB12	3.70170	0.29143	0.01200	0.01625
Ctrl vs 50 µM H_2_O_2_ + 1 µM VitB12	16.40600	0.46336	0.00200	0.00375
50 µM H_2_O_2_ vs 50 µM H_2_O_2_ + 0.010 µM VitB12	4.42320	0.17398	0.03100	0.03577
50 µM H_2_O_2_ vs 50 µM H_2_O_2_ + 1 µM VitB12	3.66560	0.14861	0.03800	0.04071
50 µM H_2_O_2_ + 0.010 µM VitB12 vs 50 µM H_2_O_2_ + 1 µM VitB12	1.38880	0.06493	0.23900	0.23900

To further investigate the observations described above, we focused on specific Raman spectral features related to lipid composition. In particular, the Raman shift at 2851 cm⁻^1^ corresponds to the symmetric CH_2_ stretching mode in lipids; the shift at 2965 cm⁻^1^ is associated with cholesteryl esters; and the shift at 3015 cm⁻^1^ is attributed to ═CH stretching in unsaturated lipids [47, 53]. Additionally, the Raman shift at 2930 cm⁻^1^, corresponding to the symmetric CH₃ stretch in proteins, was used as an internal reference for normalization in subsequent analyses [47, 53].

**Fig. 8:**
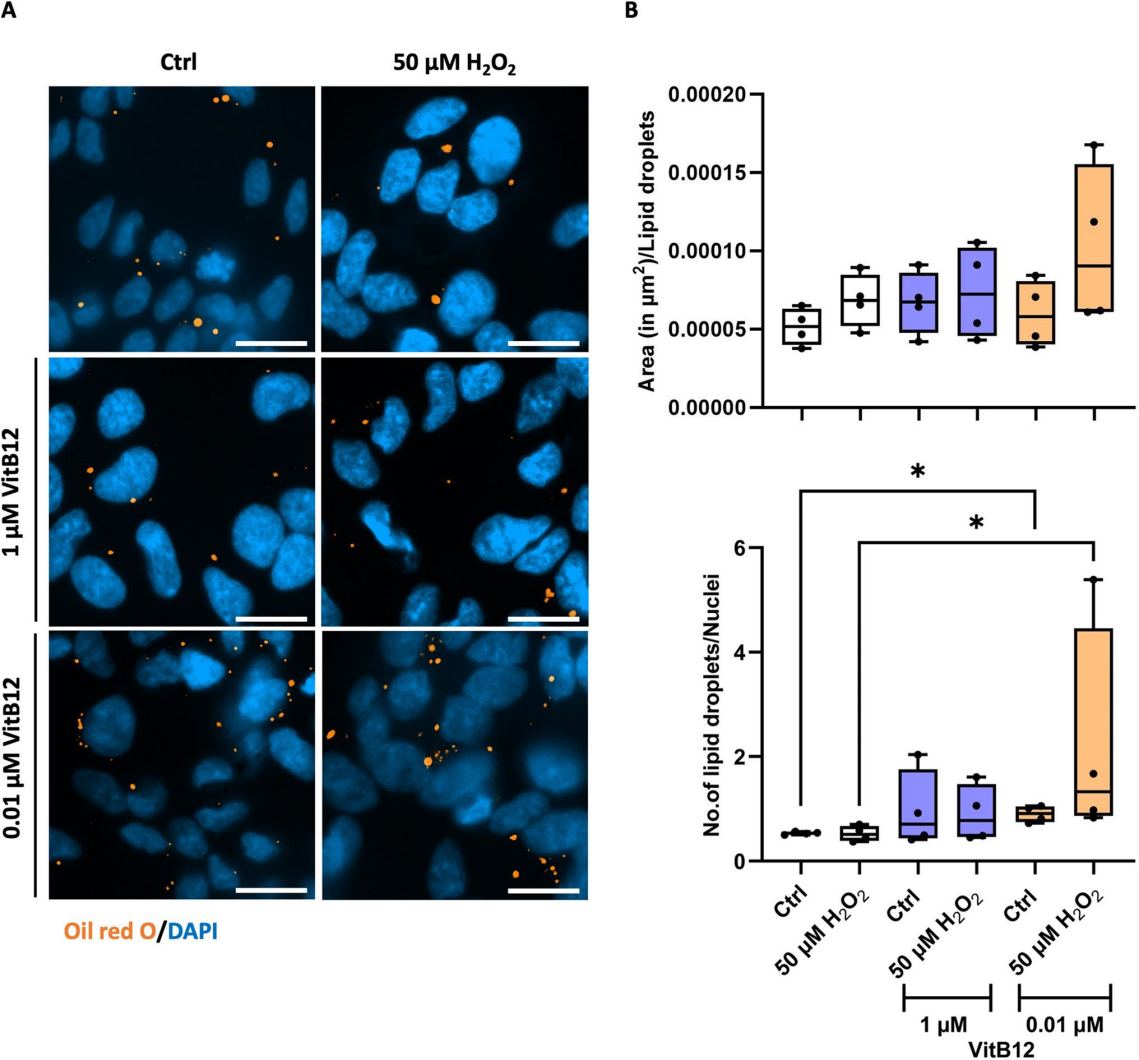
The recovery in a physiological dose of vitamin B12 after H_2_O_2_ insult promotes lipid droplet (LD) formation. **A** Microscopic images of the ORO-stained SH-SY5Y differentiated cells obtained for the different concentrations of H_2_O_2_ and VitB12 after recovery in their respective controls and the scale bar corresponds to a length of 20 µm, and **B** the quantification of the number of LDs per nuclei and the average surface area per LDs across all the treatment conditions, using the ImageJ software. The samples correspond to: Condition 1, differentiated SH-SY5Y cells in MEM containing 1% FBS; Condition 2, differentiated SH-SY5Y cells treated with 50 μM H_2_O_2_, followed by recovery in MEM containing 1% FBS; Condition 3, differentiated SH-SY5Y cells in MEM containing 1% FBS and 1 μM VitB12; Condition 4, differentiated SH-SY5Y cells treated with 50 μM H_2_O_2_, followed by recovery in MEM containing 1% FBS and 1 μM VitB12; Condition 5, differentiated SH-SY5Y cells in MEM containing 1% FBS and 0.01 μM VitB12; Condition 6, differentiated SH-SY5Y cells treated with 50 μM H_2_O_2_, followed by recovery in MEM containing 1% FBS and 0.01 μM VitB12. Data are from 4 biological replicates and are presented as mean ± SD. For the quantification of the number of LDs, a Shapiro-Wilk test was performed to identify that the data was not normally distributed. Therefore, this allowed us to perform a non-parametric statistical test, Mann-Whitney *U* test, to compare the significance between the different treatment groups. All data were considered statistically significant at *p < 0.05

The ratio of the 2851 cm⁻^1^ Raman shift (lipids) to the 2930 cm⁻^1^ shift (proteins) was used to assess the overall lipid-to-protein balance. With the exception of the comparison between non-stressed cells cultured in high-dose VitB12 and those in unsupplemented medium, no significant differences were observed, suggesting a comparable distribution of total lipid/protein content across all recovery conditions (Fig. 8S). The ratios of the 2965 cm⁻^1^ Raman shift (cholesteryl esters) to the 2930 cm⁻^1^ shift (proteins) and of the 3015 cm⁻^1^ shift (unsaturated lipids) to 2930 cm⁻^1^ showed some variation across conditions (Fig. 8S). However, to better highlight changes in relative lipid composition, we focused on the ratio of 3015 cm⁻^1^ to 2965 cm⁻^1^ (Fig. 6B). This ratio significantly increased in cells recovering with a physiological dose of VitB12 compared to those recovering without supplementation after H_2_O_2_ insult. This finding suggests a relative enrichment of unsaturated lipids over cholesteryl esters under recovery conditions supported by physiological levels of VitB12 (Fig. 6B).

Generally, under oxidative stress conditions, alterations in lipid composition can help preserve membrane fluidity and integrity, thereby supporting cell survival [54]. To better characterize this phenomenon, we focused on the two experimental groups that exhibited the most prominent differences: H_2_O_2_-insulted cells recovering in unsupplemented medium and those recovering in medium supplemented with physiological levels of VitB12.

We generated intensity maps of specific Raman shifts corresponding to the lipid signals previously mentioned. The 2851 cm⁻^1^ shift revealed distinct regions of maximal intensity (Fig. 7A, B), which, according to Pacia and team [53], are indicative of lipid droplets (LDs). This observation enabled us to investigate lipid composition in two distinct cellular compartments: the lipid-rich regions corresponding to LDs and the surrounding membrane-associated lipid areas, which primarily reflect the plasma membrane composition.

To standardize our analysis, we used Fiji software to extract intensity profiles from a segment containing two high-intensity regions at 2851 cm⁻^1^ (Fig. 7B). We then analyzed these profiles for the other Raman shifts of interest (Fig. 7C). At the LD locations, the 2930 cm⁻^1^ signal appeared consistent between the two peaks identified in each condition, suggesting a stable protein distribution under both treatment conditions. Interestingly, opposite trends were observed for the 2965 cm⁻^1^ shift (cholesteryl esters) and the 3015 cm⁻^1^ shift (unsaturated lipids) at the LD locations between the two groups. Specifically, the 3015 to 2965 cm⁻^1^ ratio decreased in the regions corresponding to LDs in cells recovering in unsupplemented medium, whereas it increased in cells recovering with a physiological dose of VitB12. This suggests a relative enrichment of unsaturated lipids over cholesterol esters within LDs under VitB12-supplemented recovery conditions (Fig. 7C). It is worth noting, however, that along the entire analyzed segment, the overall 3015 to 2965 cm⁻^1^ ratio was higher in cells recovering in unsupplemented medium compared to those recovering with a physiological dose of VitB12, thus not aligning with the SCRS analysis performed on 1-μm^2^ regions of individual cell surfaces. This discrepancy may be attributed to differences in the quantity or spatial extent of lipid droplets between the two conditions, which could disproportionately influence the overall Raman signal contribution. To evaluate this possibility, we assessed LD content directly by performing Oil Red O (ORO) staining, a fat-soluble diazo dye commonly used to label neutral lipids and quantify lipid accumulation [29].

Following ORO staining, we quantified two lipid-related parameters across all treatment groups using ImageJ software: the number of LDs per nucleus and the average surface area per LD. Our analysis revealed that, although the average surface area per LD remained unchanged, the number of LDs per nucleus increased in cells recovering with a physiological dose of VitB12 compared to the unsupplemented condition (Fig. 8A, B). It is plausible that this increased abundance contributes additively to the overall Raman signal. Consequently, the 3015 to 2965 cm^−1^ ratio measured over broader cell areas by SCRS is elevated due to the cumulative signal originating from a larger population of LDs.

It is important to note that neuronal cells are typically not considered energy-storing cells, as they lack significant glycogen or LD reserves [55]. The combination of an enrichment in unsaturated fatty acids and an increase in LD formation following the physiological dose of VitB12 supplementation presents several interesting possibilities. Under conditions of oxidative stress, an increased unsaturated fatty acid content in LDs could reflect an adaptive response and may contribute to buffering oxidative stress, thereby mitigating H_2_O_2_-induced damage, but also by replenishing cell membranes with more fluid lipids, thereby helping to preserve membrane integrity and fluidity [56].

## Conclusions

Here, we used an in vitro neural-like model of differentiated SH-SY5Y cells exposed to hydrogen peroxide (H_2_O_2_) to investigate the effects of physiological (0.01 µM) versus high (1 µM) doses of VitB12 on neural recovery. Our results demonstrate that a physiological dose significantly enhances cell viability and neurite outgrowth compared to a high dose. At 2 h of recovery, notably, this dose triggered earlier activation of antioxidant gene expression and increased the JC-1 R/G ratio (ΔΨm), suggesting a more rapid cellular response to H_2_O_2_ insult. In contrast, high-dose VitB12 markedly suppressed DCFDA positivity after H_2_O_2_, underscoring the importance of maintaining an appropriate redox balance. At 24 h of recovery, metabolic analysis (exploratory NMR endo-metabolomics) suggested reduced intracellular succinate levels with the physiological dose. Consistently, mitochondrial network metrics indicated remodeling toward a more interconnected morphology, reflected by greater mean branch length in the mitochondrial network. Taken together, these convergent observations across orthogonal approaches support a dose-dependent mitochondrial adaptation during recovery, while acknowledging that the NMR layer is exploratory and will benefit from future targeted validation. This was accompanied by lipid remodeling, as detected by single-cell Raman spectroscopy (SCRS), indicating increased levels of unsaturated lipids. In parallel, Oil Red O (ORO) staining revealed a higher number of lipid droplets (LDs), suggesting active lipid-based stress responses. Importantly, the accumulation of unsaturated lipids and LDs may represent additional late-stage defense mechanisms against H_2_O_2_-induced oxidative damage. These findings should be interpreted considering model- and time-related limitations (RA-differentiated SH-SY5Y cells and predominantly 24-h recovery endpoints) and will require validation in primary neurons/in vivo with expanded time-course analyses; mechanistic causality will further benefit from targeted genetic perturbations of key antioxidant and lipid-droplet regulators.

In summary, our findings indicate that a physiological dose of VitB12 orchestrates a temporally coordinated response to oxidative stress, characterized by the early activation of antioxidant defenses—unlike the delayed response observed with the high dose—and followed, at later time points, by mitochondrial and lipid-mediated adaptive mechanisms. This aligns with previous reports showing that VitB12 can support neuronal structural recovery and mitigate oxidative injury [10–14]. Similar beneficial associations between VitB12 status/supplementation and cognitive or neuroprotective outcomes have also been reported in vivo, supporting the broader relevance of VitB12 for neuronal resilience [10–12]. Importantly, our study extends this literature by directly comparing a physiological (low-nanomolar) dose with a higher dose and demonstrating that the timing and nature of the recovery program are dose-dependent within the same experimental framework. It is important to note that studies have shown that VitB12 at a high dose can have very specific effect; for instance, it can modulate LRRK2 kinase activity through allosteric regulation, conferring neuroprotection in models of Parkinson’s disease [15, 57]. This suggests that, while high doses of VitB12 may engage specific signaling pathways contributing to neuroprotection, they may also affect distinct metabolic routes that are not conducive to recovery from oxidative stress, particularly that induced by H_2_O_2_, highlighting the complexity of dose-dependent effects (reviewed in [15]). Although these findings should be interpreted considering model- and time-related limitations (RA-differentiated SH-SY5Y cells and 2–24-h recovery endpoints) and will require validation in primary neurons/in vivo with expanded time-course analyses, they provide a promising rationale that different VitB12 doses may activate distinct protective mechanisms, underscoring the importance of tailoring the choice between high and low doses to the type and context of cellular damage, as dose requirements may be context-dependent in redox-sensitive recovery settings.

## Supplementary Information

Below is the link to the electronic supplementary material.

ESM 1 (DOCX 5.28 MB)

ESM 2 (DOCX 807 KB)

## Data Availability

The authors declare that the data supporting the findings of this study are available within the paper and its Supplementary Information files. Should any raw data files be needed in another format they are available from the corresponding author upon reasonable request.
