# Supplementary material for: Less Is More: A Physiological Dose of Vitamin B12 Enhances Neural Recovery Compared to a High Dose in an H₂O₂-Stressed SH-SY5Y Neural-Like Cell Model
Source: Mol Neurobiol. 2026 Apr 17;63(1):569. doi: 10.1007/s12035-026-05841-9 (PMC13090186; doi:10.1007/s12035-026-05841-9)
Supplement: Supplementary file 1 — (DOCX 5.28 MB) [file 12035_2026_5841_MOESM1_ESM.docx]

Supplementary Materials

**Figure 1S. RA-differentiated SH-SY5Y as a neural-like cell model.** Human SH-SY5Y cells exposed to 10 µM of retinoic acid (RA) express neuron-like markers and exhibit neurite outgrowth processes (Juan et al., 2014; Lopes et al., 2010). We characterized the neural (Synaptophysin, SYP, and Growth Associated Protein 43, GAP-43) (Juan et al., 2014; Lopes et al., 2010) and proliferative (Proliferating Cell Nuclear Antigen, PCNA) (Kovalevich & Langford, 2013) protein markers by WB at 3 days; and assessed the length of the neurite outgrowth processes at 3, 6, and 9 days using the NeuronJ plugin of the ImageJ software (Pemberton et al., 2018) (Figure 1S A-D). After 3 days of RA treatment, the proliferation marker PCNA was significantly reduced, while SYP increased. Altogether, this clearly demonstrated the change from proliferative phenotype to neuron-like phenotype (Kovalevich & Langford, 2013; Lopes et al., 2010). GAP-43 modulates actin dynamics (Chung et al., 2020), playing a crucial role in axonal growth and regeneration (Juan et al., 2014), as well as in neuroprotection and synaptic maturation (Romeo-Guitart et al., 2018). Specifically, GAP-43 promotes growth cone motility and axon outgrowth. Axonal elongation leads to a reduction in GAP-43 levels which are kept aiming at maintaining turnover (Chung et al., 2020). In undifferentiated cells, GAP-43 accumulates, and as differentiation progresses following RA treatment, neurite elongation occurs due to actin assembly and the GAP-43 protein level decreases (Figure 1S C-D). This reduction in GAP-43 may be associated with neurite elongation (Holahan, 2015), and thereby the recovery process as well. Regarding the neurites, we observed a difference in their lengths after 3 days of RA treatment, wherein a plateau was attained after 6 and 9 days of differentiation. This suggests that there exists no further potential for neurite elongation beyond 6 days, and any treatments administered to the cells differentiated for 6 or 9 days would not reveal any additional positive effects on the neurite length. Considering this, along with the neural and proliferative markers analyzed, we concluded that a 3-day treatment with 10 µM RA induces a suitable differentiation state that is appropriate for our research objectives.

The protein levels of CD320 were also evaluated, since the CD320 receptor is responsible for the cellular uptake of VitB12 (reviewed in (Mathew et al., 2024)), and although we observe a reduction after the differentiation, it was still expressed (Figure 1S C-D).**(A)** Bright-field microscopic images of undifferentiated SH-SY5Y cells and SH-SY5Y cells differentiated with RA for 3 days, 6 days, and 9 days. Und – Undifferentiated SH-SY5Y cells in MEM + 10% FBS; Diff – RA-differentiated SH-SY5Y cells in MEM + 1% FBS and 10 µM RA. Scale bar corresponds to a length of 50 µm. **(B)** The quantification of the length of the neurite outgrowth processes (in µm) obtained for the undifferentiated and differentiated SH-SY5Y cells (3-days, 6-days and 9-days). **(C)** The western blots of all the characterized protein markers, including PCNA, SYP, GAP-43, CD320 and β-Actin. β-Actin was employed as the internal reference protein. The samples correspond to: Lane 1 – Undifferentiated SH-SY5Y cells; Lane 2 – 3-day differentiated SH-SY5Y cells. **(D)** The densitometric analysis of the above-mentioned markers were performed by evaluating the protein expression levels from the western blot analysis of the protein bands normalized with the bands of β-Actin. Data are from 9 biological replicates for neurites and 3 biological replicates for WB and is presented as mean ± standard deviation (SD). Statistical significance was determined using a one-way ANOVA test, followed by Tukey's test, or t-test: *p < 0.05, **p < 0.01, ***p < 0.001, ***p < 0.0001

Figure 2S. **EDS detects intracellular cobalt following VitB12 supplementation.**

RA-differentiated human SH-SY5Y cells were analyzed by EDS to detect intracellular cobalt (Co) as a proxy for cellular entry of cobalamin (VitB12), the only known essential biological reservoir of Co in animals/humans (EFSA Panel on Additives and Products or Substances used in Animal Feed (FEEDAP), 2009). Semi-quantitative Co levels are reported as Co Lα1 counts (cps/eV) and as relative atomic fraction (at.%) among the detected elements (C, N, O, Co, Na, Si). Because intracellular Co is expected to be extremely low, measurements were performed under optimized, high-sensitivity acquisition conditions close to the detection limit (high-count acquisition settings; Co Lα1 line at ~0.77 keV; low accelerating voltage to maximize electron–matter interaction and X-ray generation).

Figure 3S**. The effect of the physiological dose of vitamin B12 on the expression levels of Caspase-3.** The western blots of Pro-caspase 3, Caspase-3 and β-Actin. β-Actin was employed as the internal reference protein. The samples correspond to: Lane 1 – Differentiated SH-SY5Y cells in MEM containing 1% FBS; Lane 2 – Differentiated SH-SY5Y cells treated with 50 μM H_2_O_2_, followed by recovery in MEM containing 1% FBS; Lane 3 – Differentiated SH-SY5Y cells in MEM containing 1% FBS and 1 μM VitB12; Lane 4 – Differentiated SH-SY5Y cells treated with 50 μM H_2_O_2_, followed by recovery in MEM containing 1% FBS and 1 μM VitB12; Lane 5 – Differentiated SH-SY5Y cells in MEM containing 1% FBS and 0.01 μM VitB12; Lane 6 – Differentiated SH-SY5Y cells treated with 50 μM H_2_O_2_, followed by recovery in MEM containing 1% FBS and 0.01 μM VitB12. The densitometric analysis was performed by evaluating the protein expression levels from the protein bands, and ratio of Caspase-3 to Pro-caspase-3 was quantified.

Figure 4S. **Assessment of early cytosolic ROS-dependent redox status (DCFDA) during recovery (2 h) after H₂O₂.** (A) Representative fluorescence images of DCFDA staining acquired at 2 h recovery (200×). (B) Quantification of the percentage of DCFDA-positive cells per condition. Positive cells were defined using an internal, experiment-specific threshold derived from the H₂O₂ condition. Quantitative image analysis was performed using Fiji/ImageJ software. Statistical significance was determined using one-way ANOVA followed by Student’s t-test: *p

$\leq$ 0.05, **p < 0.01, ***p < 0.001, ****p < 0.0001.

Figure 5S. **Assessment of mitochondrial membrane potential (ΔΨm) using JC-1 during recovery (2 h) after H₂O₂ insult.** (A) Representative JC-1 green-channel images (monomer signal) and red-channel images (aggregate signal) acquired at 2 h recovery (200×). (B) Quantification of the JC-1 red/green fluorescence ratio (R/G). Quantitative image analysis was performed using Fiji/ImageJ software. Statistical significance was determined using one-way ANOVA followed by Student’s t-test: *p

$\leq$ 0.05, **p < 0.01, ***p < 0.001, ****p < 0.0001.

**Figure 6S. NMR endo-metabolome analysis and MetaboAnalyst.ca result.** (A) The metabolic profile alterations (differences in the metabolites) from the endo-metabolome analysis revealed by a PCA plot showing the distribution of metabolites with different colors indicating different experimental groups. PC1 represents amino acid metabolism and explains 49.5%, and PC2 represents energy metabolism accounts for 17.3% of the total variance. The conditions correspond to: Condition 1 – Differentiated SH-SY5Y cells in MEM containing 1% FBS; Condition 2 – Differentiated SH-SY5Y cells treated with 50 μM H_2_O_2_, followed by recovery in MEM containing 1% FBS; Condition 3 – Differentiated SH-SY5Y cells in MEM containing 1% FBS and 1 μM VitB12; Condition 4 – Differentiated SH-SY5Y cells treated with 50 μM H_2_O_2_, followed by recovery in MEM containing 1% FBS and 1 μM VitB12; Condition 5 – Differentiated SH-SY5Y cells in MEM containing 1% FBS and 0.01 μM VitB12;  Condition 6 – Differentiated SH-SY5Y cells treated with 50 μM H_2_O_2_, followed by recovery in MEM containing 1% FBS and 0.01 μM VitB12. **(B)** A pathway analysis from MetaboAnalyst.ca, employing the metabolites that were altered after H_2_O_2_ insult and recovered in unsupplemented vs H_2_O_2_-insulted cells recovered in VitB12 physiological dose media. The metabolites included in the analysis were C01042-N-acetyl L-aspartate, C00041-Alanine, C03912-(S)-1-Pyrroline-5-carboxylate, C00158-Citrate, C00049-L-Aspartic acid, C02362-Gamma amino oxaloacetate, C00402-L-Glutamate, C12270-2-Oxo glutarate, C00022-L-Glutamine, C00940-L-Glutamic acid, C00025-L-Glutamate, C20775-2-Oxoglutarate, C00152-L-Asparagine, C03406-L-Argininosuccinate, C03794-N-Carbamoyl-L-aspartate, C00438- Carbamoyl-L-aspartate, C00036-L-Glutamine, C20776-N-carbamoyl-L-aspartate, C00026-Alpha ketoglutarate, C00334-L-Glutamate, C00064-L-Glutamine, C00232-L-Glutamine, C00014-Ammonia, C00352-L-Glutamate, C03090-L-Glutamine, C00042-Succinate and C00169-Carbamoyl phosphate. Data are obtained from 3 biological replicates and are presented as mean ± SD.


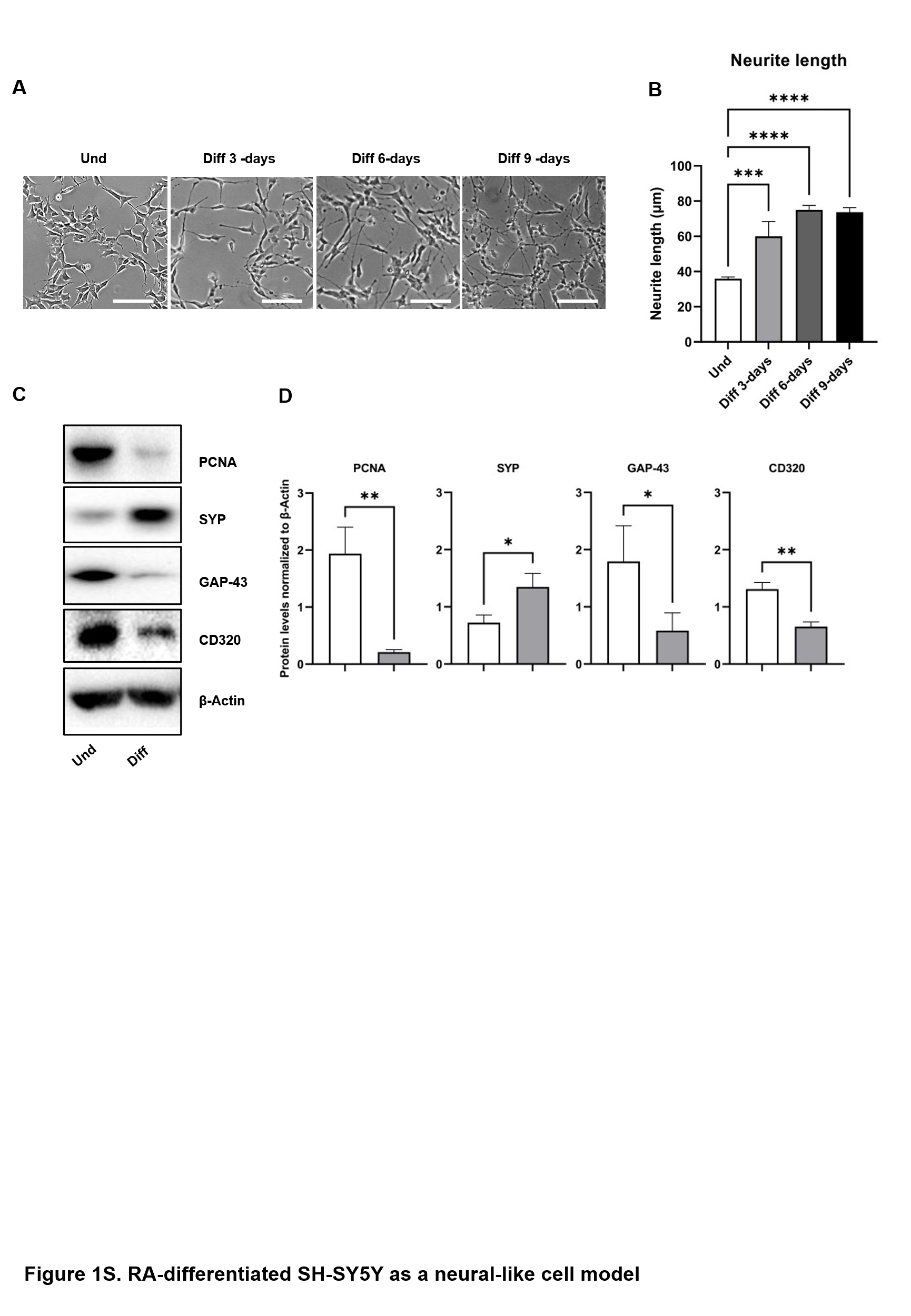


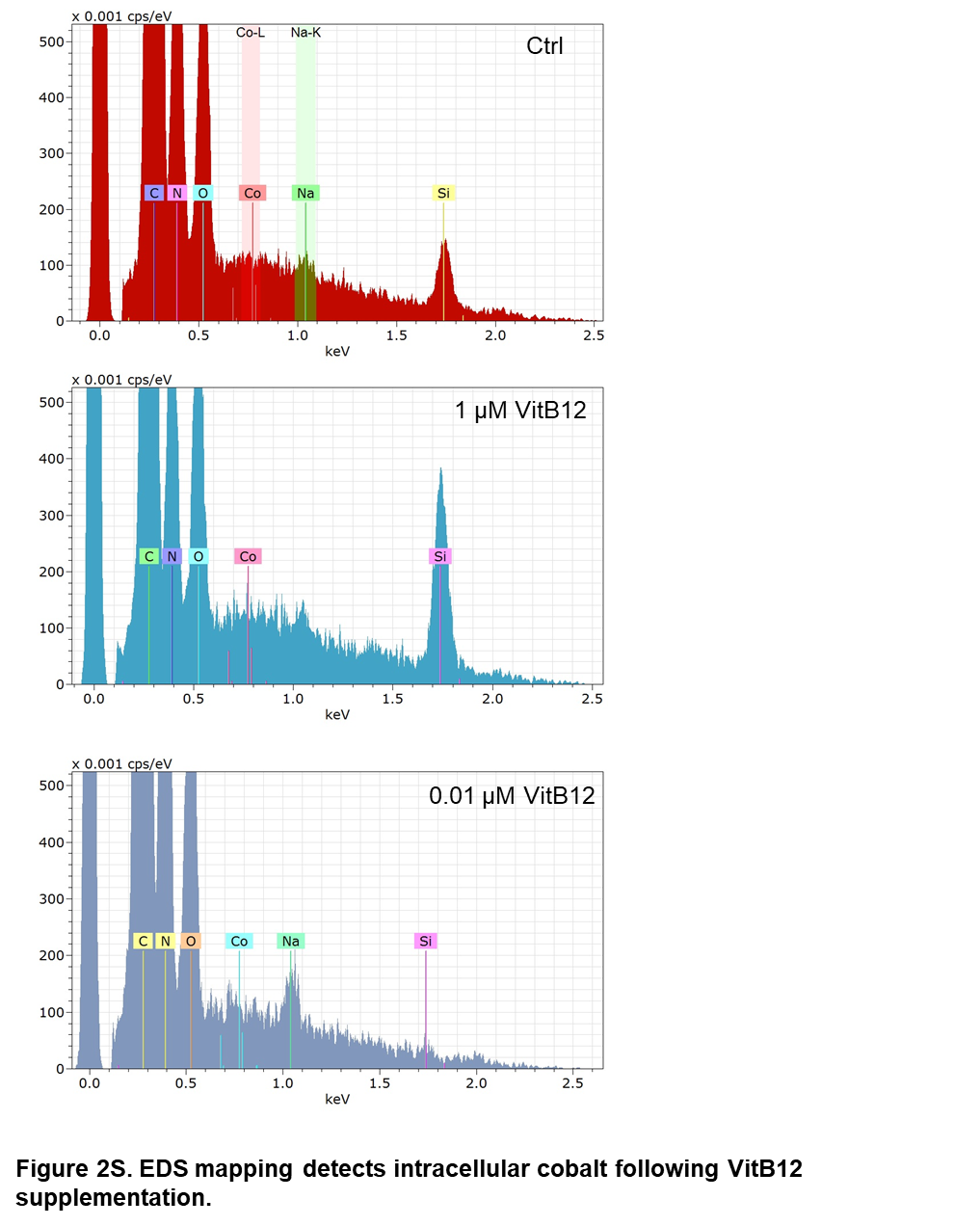


| **Table 1S. Normalized wt.% of Co with respect to N, O, C, Si, and Na** | |
| --- | --- |
| Treatment | wt.% |
| Ctrl | 0 |
| 1 µM | 0.81 |
| 0.01 µM | 0.06 |


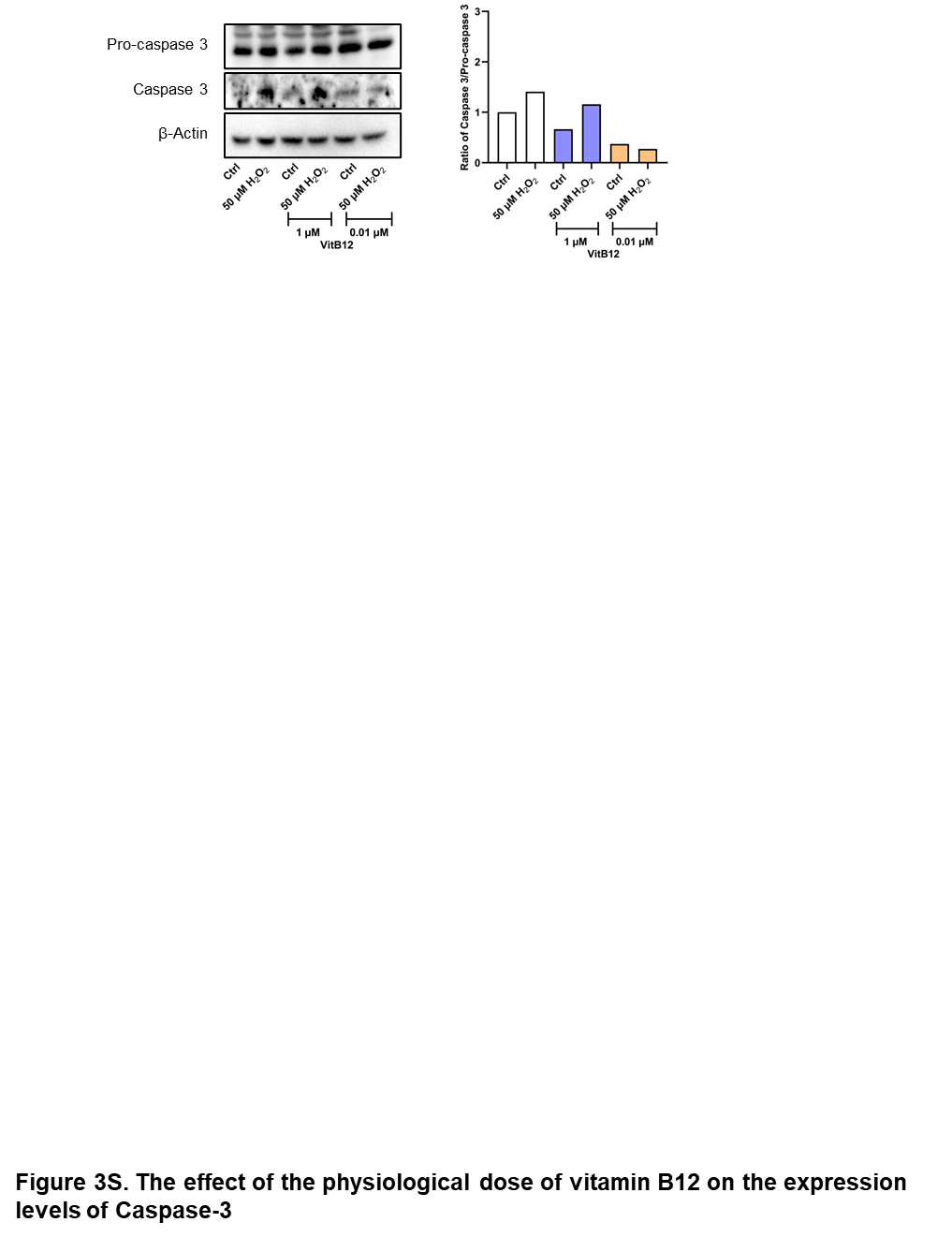


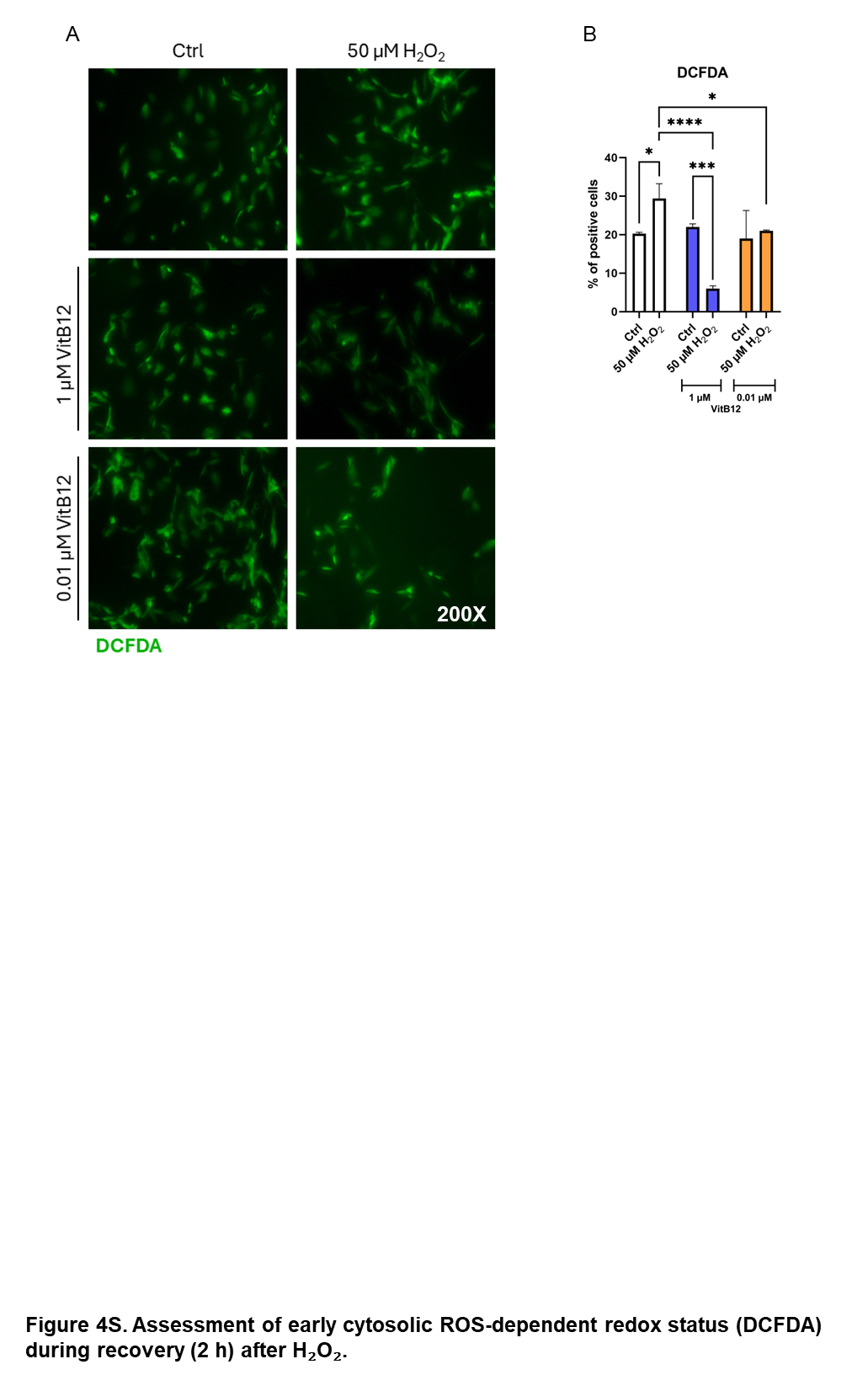


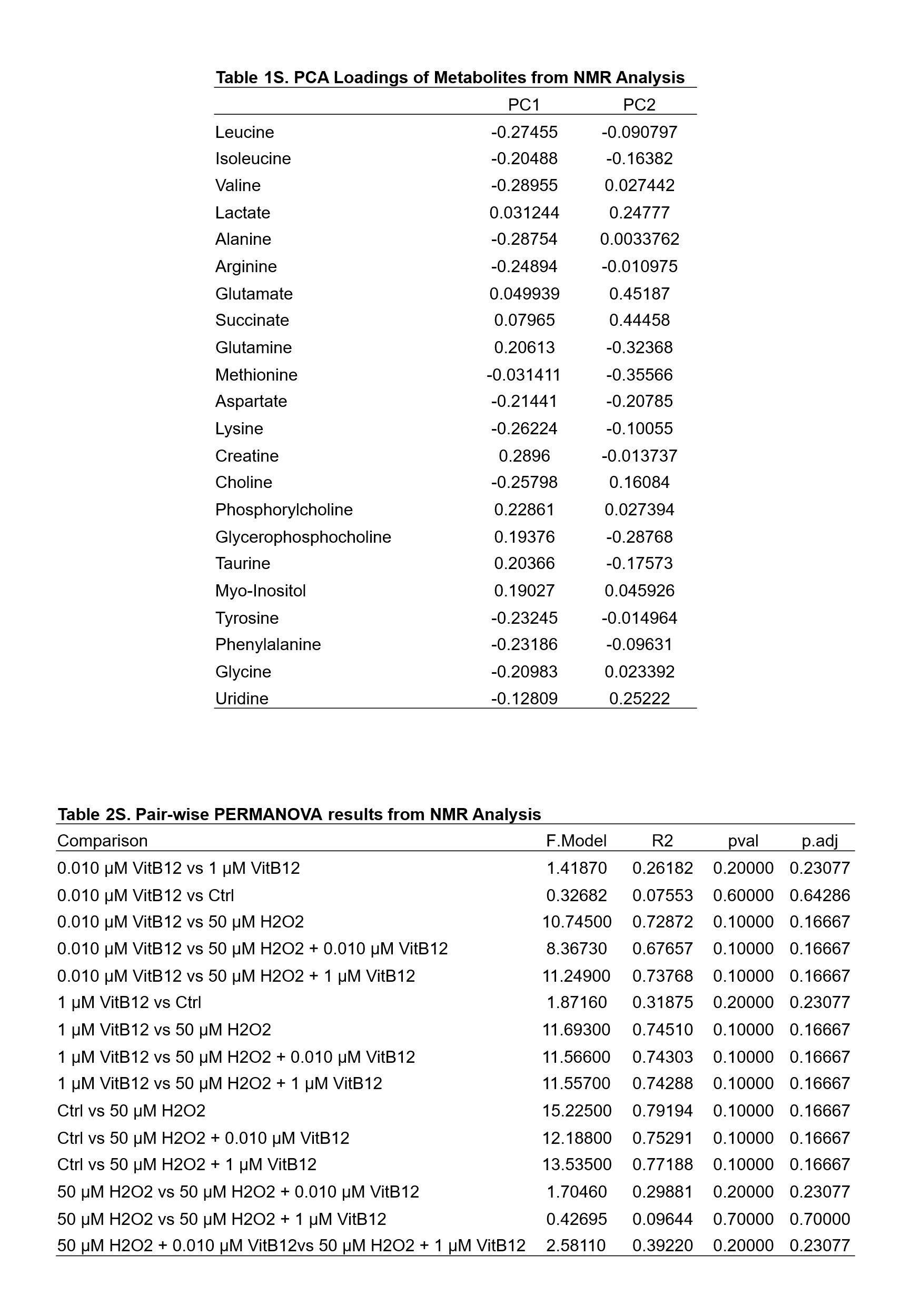


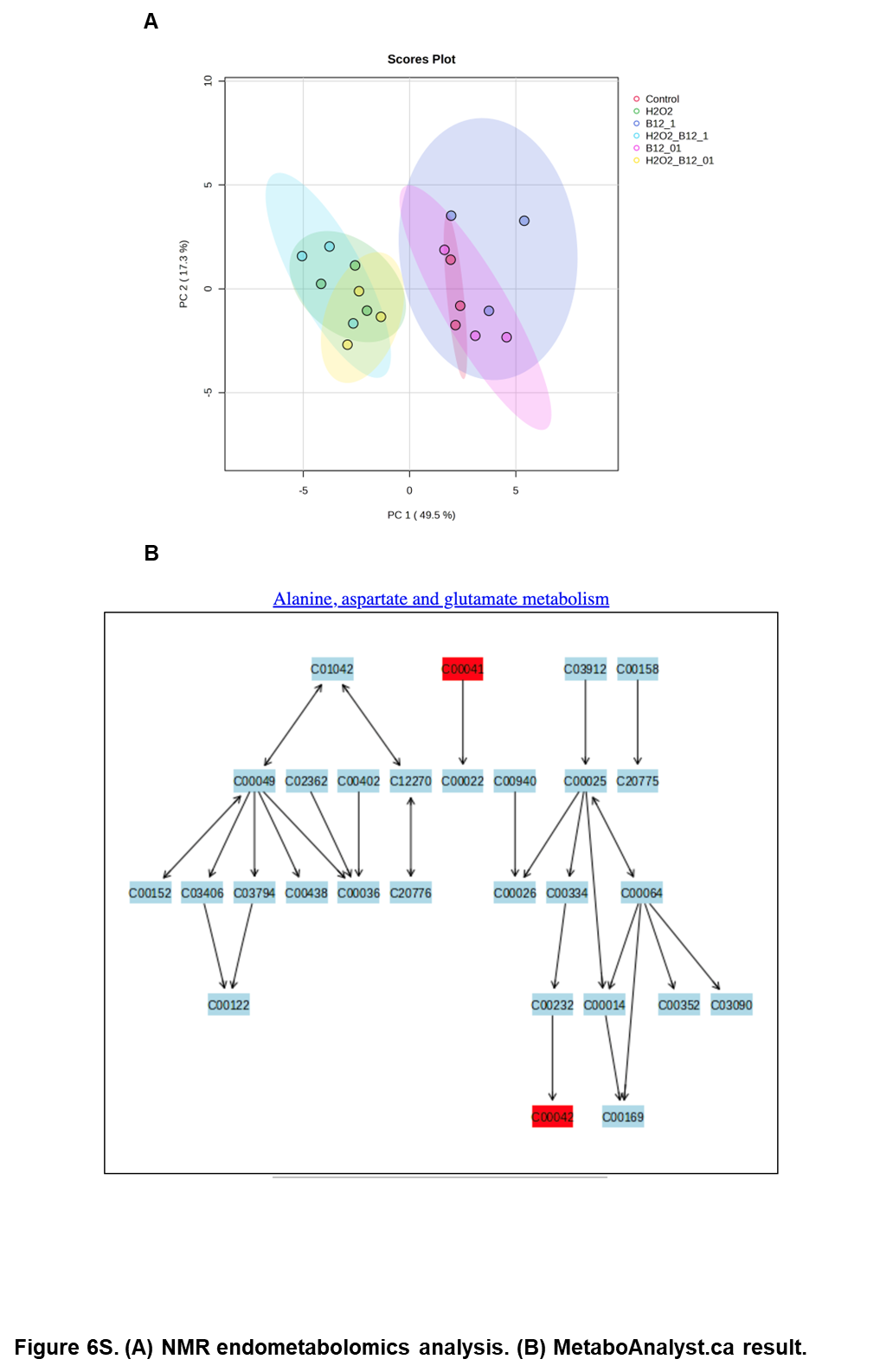


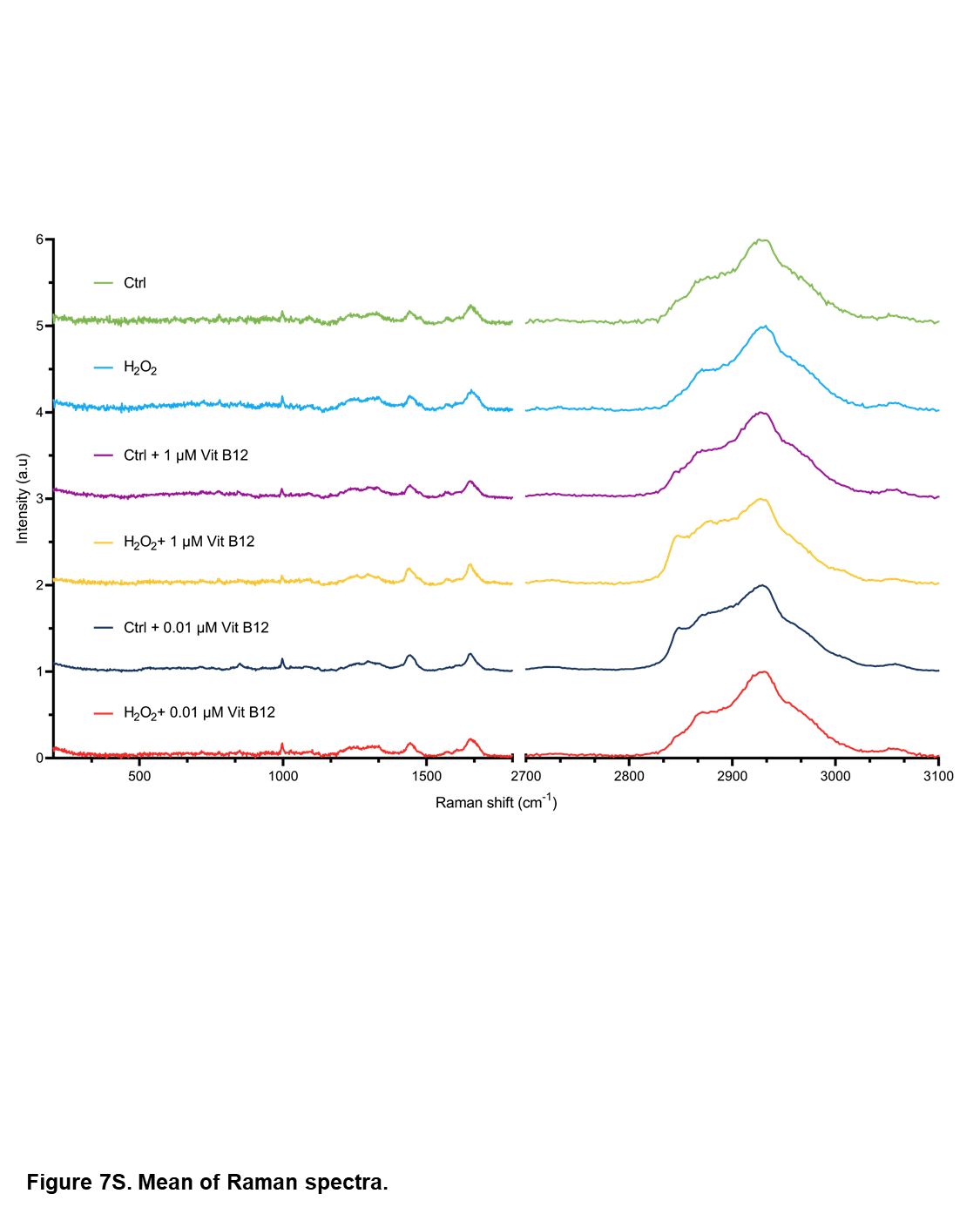


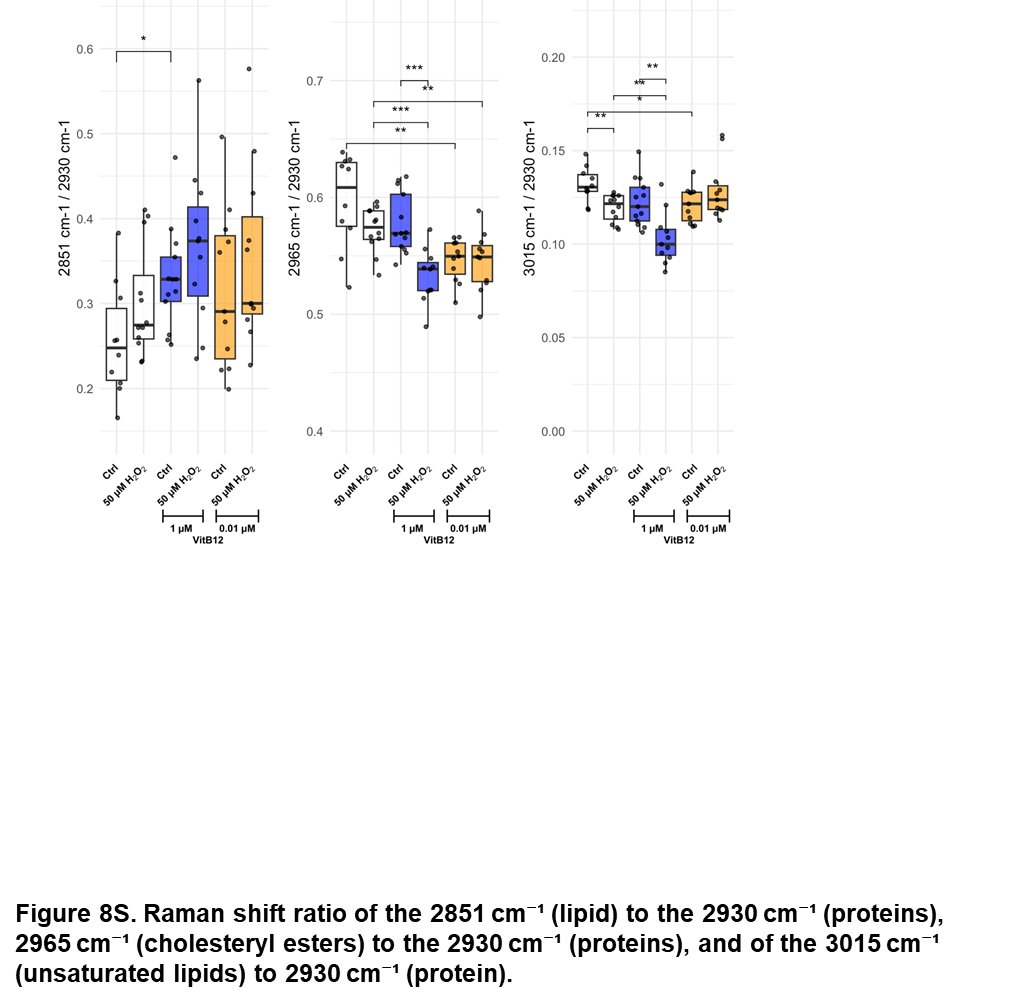


**References**

Chung, D., Shum, A., & Caraveo, G. (2020). GAP-43 and BASP1 in Axon Regeneration: Implications for the Treatment of Neurodegenerative Diseases. In *Frontiers in Cell and Developmental Biology* (Vol. 8). Frontiers Media S.A. https://doi.org/10.3389/fcell.2020.567537

EFSA Panel on Additives and Products or Substances used in Animal Feed (FEEDAP). (2009). Scientific Opinion on the use of cobalt compounds as additives in animal nutrition. *EFSA Journal*, *7*(12), 1383. https://doi.org/10.2903/j.efsa.2009.1383

Holahan, M. (2015). GAP-43 in synaptic plasticity: molecular perspectives. *Research and Reports in Biochemistry*, 137. https://doi.org/10.2147/rrbc.s73846

Juan, W. S., Huang, S. Y., Chang, C. C., Hung, Y. C., Lin, Y. W., Chen, T. Y., Lee, A. H., Lee, A. C., Wu, T. S., & Lee, E. J. (2014). Melatonin improves neuroplasticity by upregulating the growth-associated protein-43 (GAP-43) and NMDAR postsynaptic density-95 (PSD-95) proteins in cultured neurons exposed to glutamate excitotoxicity and in rats subjected to transient focal cerebral ischemia even during a long-term recovery period. *Journal of Pineal Research*, *56*(2), 213–223. https://doi.org/10.1111/jpi.12114

Kovalevich, J., & Langford, D. (2013). Considerations for the use of SH-SY5Y neuroblastoma cells in neurobiology. *Methods in Molecular Biology*, *1078*, 9–21. https://doi.org/10.1007/978-1-62703-640-5_2

Lopes, F. M., Schröder, R., Júnior, M. L. C. da F., Zanotto-Filho, A., Müller, C. B., Pires, A. S., Meurer, R. T., Colpo, G. D., Gelain, D. P., Kapczinski, F., Moreira, J. C. F., Fernandes, M. da C., & Klamt, F. (2010). Comparison between proliferative and neuron-like SH-SY5Y cells as an in vitro model for Parkinson disease studies. *Brain Research*, *1337*, 85–94. https://doi.org/10.1016/j.brainres.2010.03.102

Mathew, A. R., Di Matteo, G., La Rosa, P., Barbati, S. A., Mannina, L., Moreno, S., Tata, A. M., Cavallucci, V., & Fidaleo, M. (2024). Vitamin B12 Deficiency and the Nervous System: Beyond Metabolic Decompensation—Comparing Biological Models and Gaining New Insights into Molecular and Cellular Mechanisms. In *International Journal of Molecular Sciences* (Vol. 25, Number 1). Multidisciplinary Digital Publishing Institute (MDPI). https://doi.org/10.3390/ijms25010590

Pemberton, K., Mersman, B., & Xu, F. (2018). ARTICLE Using ImageJ to Assess Neurite Outgrowth in Mammalian Cell Cultures: Research Data Quantification Exercises in Undergraduate Neuroscience Lab. In *The Journal of Undergraduate Neuroscience Education* (Vol. 16, Number 2). www.funjournal.org

Romeo-Guitart, D., Forés, J., Herrando-Grabulosa, M., Valls, R., Leiva-Rodríguez, T., Galea, E., González-Pérez, F., Navarro, X., Petegnief, V., Bosch, A., Coma, M., Mas, J. M., & Casas, C. (2018). Neuroprotective Drug for Nerve Trauma Revealed Using Artificial Intelligence. *Scientific Reports*, *8*(1). https://doi.org/10.1038/s41598-018-19767-3
