# Supplementary material for: Less Is More: A Physiological Dose of Vitamin B12 Enhances Neural Recovery Compared to a High Dose in an H₂O₂-Stressed SH-SY5Y Neural-Like Cell Model
Source: Mol Neurobiol. 2026 Apr 17;63(1):569. doi: 10.1007/s12035-026-05841-9 (PMC13090186; doi:10.1007/s12035-026-05841-9)
Supplement: Supplementary file 2 — (DOCX 807 KB) [file 12035_2026_5841_MOESM2_ESM.pdf]

Full Uncropped Blots Images

Series 1

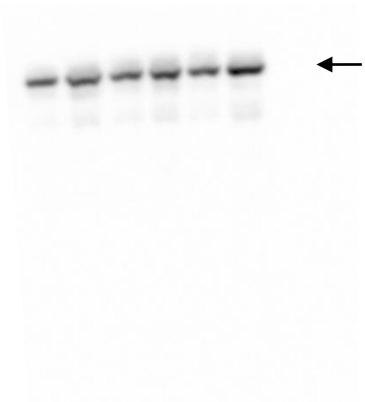

PARP1, long form, low exposition

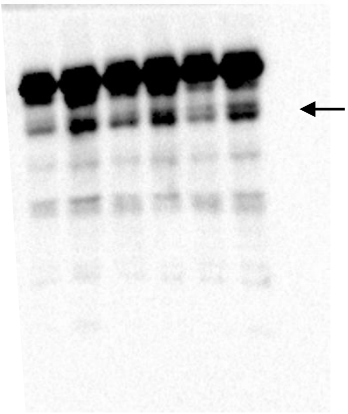

PARP1, long form, high exposition

Cleaved PARP1, long form, high exposition

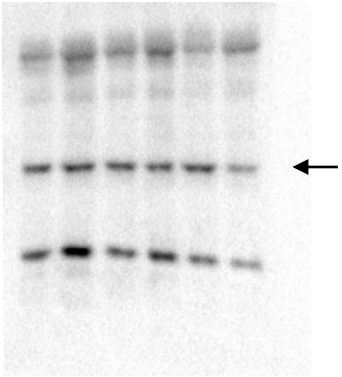

Catalase

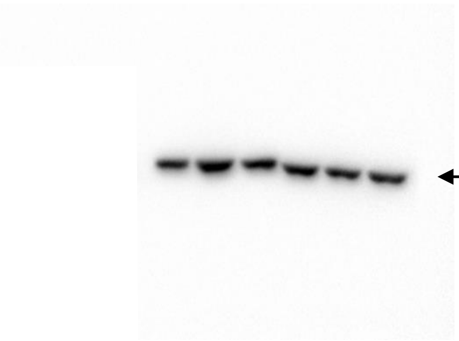

ActinB
(the membrane was cut before incubation with the antibody).

Lane 1 (Ctrl), lane 2 (50 uM H2O2 recovering 24hrs), lane 3 (1 uM VitB12 treated for 24hrs), lane 4 (50 uM H2O2 + 1 uM VitB12 recovering 24hrs), lane 5 (0.01 uM VitB12 treated for 24hrs), lane 6 (50 uM H2O2 + 0.01 uM VitB12 recovering 24hrs).

Series 1

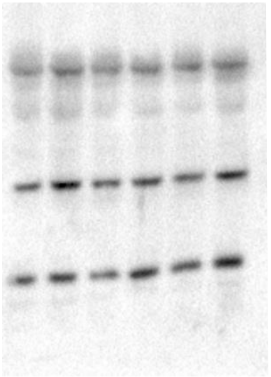

Catalase

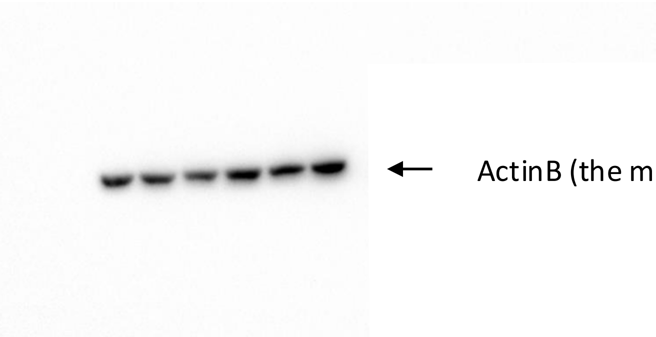

ActinB (the membrane was cut before incubation with the antibody).

Lane 1 (Ctrl), lane 2 (50 uM H2O2 recovering 2hrs), lane 3 (1 uM VitB12 treated for 2hrs), lane 4 (50 uM H2O2 + 1 uM VitB12 recovering 2hrs), lane 5 (0.01 uM VitB12 treated for 2hrs), lane 6 (50 uM H2O2 + 0.01 uM VitB12 recovering 2hrs).

Series 1

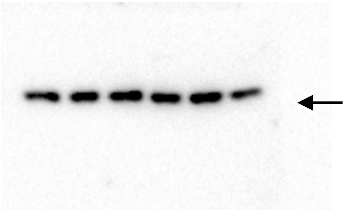

PCNA (the membrane was cut before incubation with the antibody)

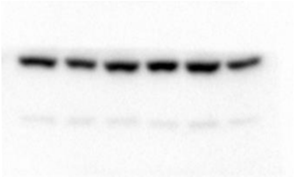

ActinB (the membrane was cut before incubation with the antibody).

Lane 1 (Ctrl), lane 2 (50 uM H2O2 recovering 24hrs), lane 3 (1 uM VitB12 treated for 24hrs), lane 4 (50 uM H2O2 + 1 uM VitB12 recovering 24hrs), lane 5 (0.01 uM VitB12 treated for 24hrs), lane 6 (50 uM H2O2 + 0.01 uM VitB12 recovering 24hrs).

Series 1

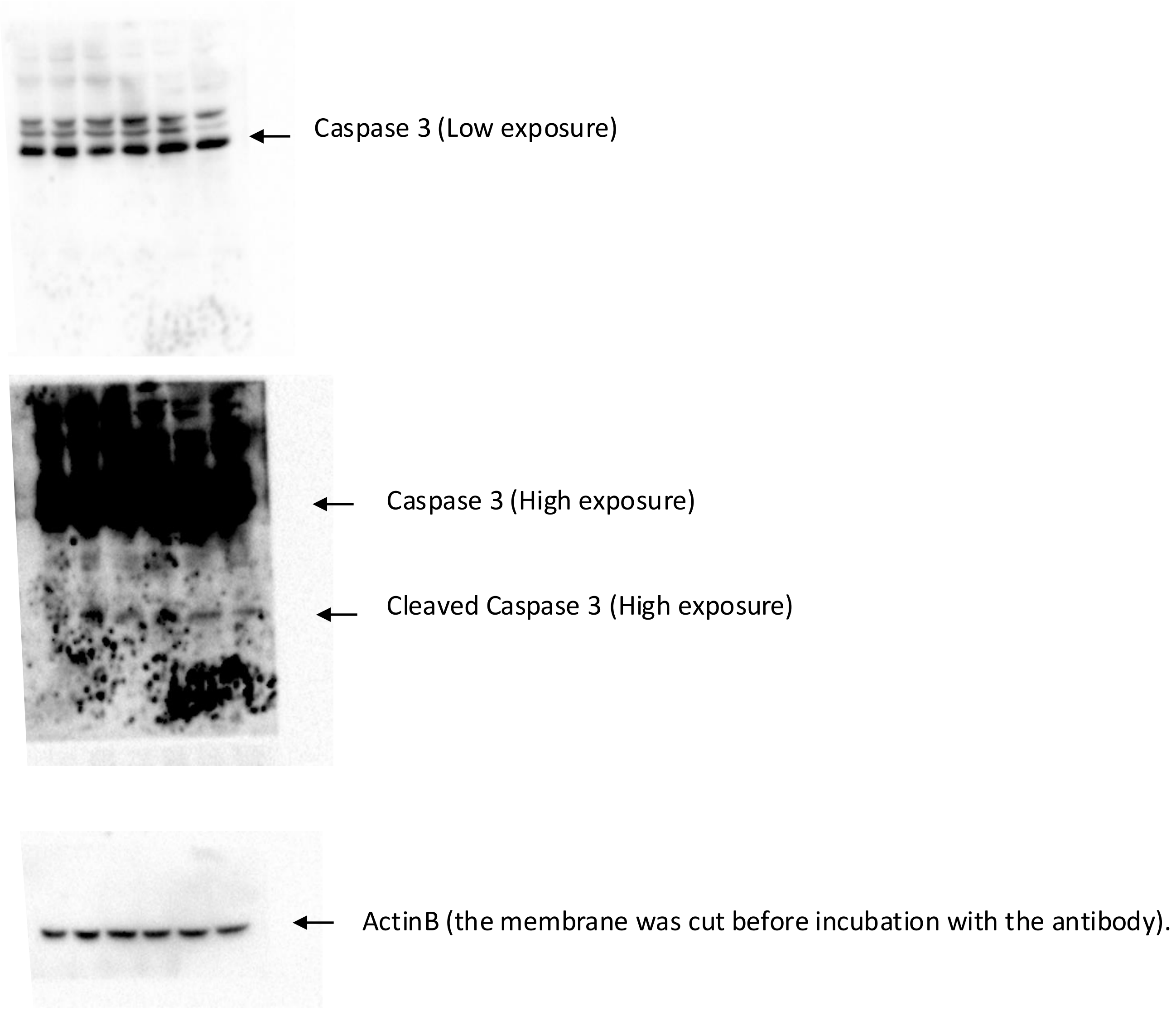

Lane 1 (Ctrl), lane 2 (50 uM H2O2 recovering 24hrs), lane 3 (1 uM VitB12 treated for 24hrs), lane 4 (50 uM H2O2 + 1 uM VitB12 recovering 24hrs), lane 5 (0.01 uM VitB12 treated for 24hrs), lane 6 (50 uM H2O2 + 0.01 uM VitB12 recovering 24hrs).

Series 1

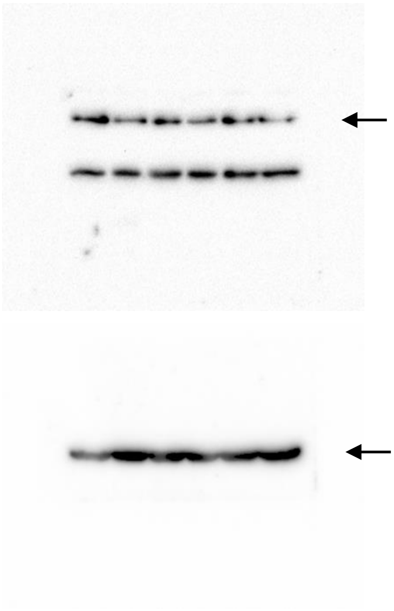

GAP-43 (the membrane was cut before incubation with the antibody).

ActinB (the membrane was cut before incubation with the antibody).

Lane 1 (Ctrl), lane 2 (50 uM H2O2 recovering 24hrs), lane 3 (1 uM VitB12 treated for 24hrs), lane 4 (50 uM H2O2 + 1 uM VitB12 recovering 24hrs), lane 5 (0.01 uM VitB12 treated for 24hrs), lane 6 (50 uM H2O2 + 0.01 uM VitB12 recovering 24hrs).

Series 2

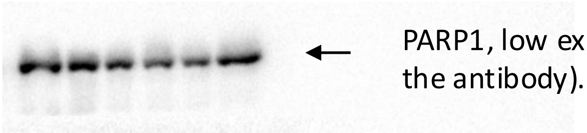

PARP1, low exposition (the membrane was cut before incubation with the antibody).

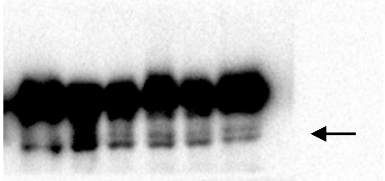

PARP1 cleaved, high exposition (the membrane was cut before incubation with the antibody).

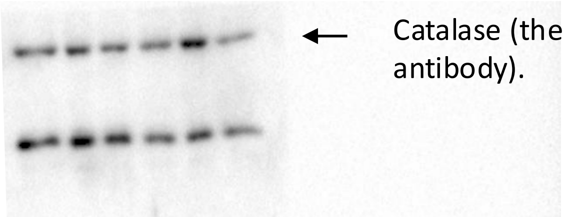

Catalase (the membrane was cut before incubation with the antibody).

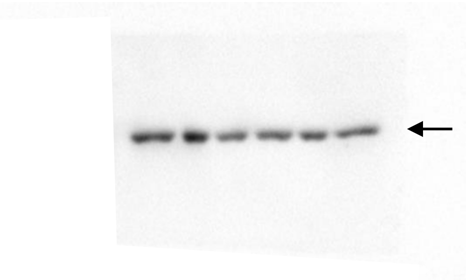

Actin (the membrane was cut before incubation with the antibody).

Lane 1 (Ctrl), lane 2 (50 uM H_2O_2 recovering 24hrs), lane 3 (1 uM VitB12 treated for 24hrs), lane 4 (50 uM H_2O_2 + 1 uM VitB12 recovering 24hrs), lane 5 (0.01 uM VitB12 treated for 24hrs), lane 6 (50 uM H_2O_2 + 0.01 uM VitB12 recovering 24hrs).

Series 2

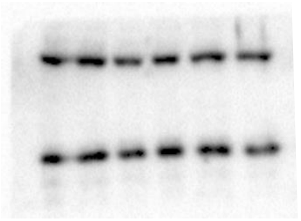

Catalase
(the membrane was cut before incubation with the antibody).

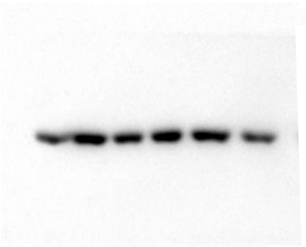

Actin (the membrane was cut before incubation with the antibody).

Lane 1 (Ctrl), lane 2 (50 uM H2O2 recovering 2hrs), lane 3 (1 uM VitB12 treated for 2hrs), lane 4 (50 uM H2O2 + 1 uM VitB12 recovering 2hrs), lane 5 (0.01 uM VitB12 treated for 2hrs), lane 6 (50 uM H2O2 + 0.01 uM VitB12 recovering 2hrs).

Series 2

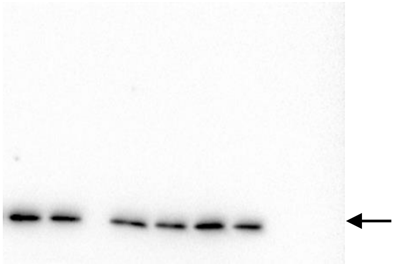

PCNA (the membrane was cut before incubation with the antibody).

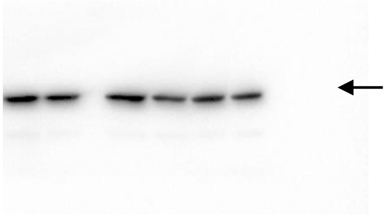

Actin (the membrane was cut before incubation with the antibody).

Lane 1 (Ctrl), lane 2 (50 uM H2O2 recovering 24hrs), lane 3 (1 uM VitB12 treated for 24hrs), lane 4 (50 uM H2O2 + 1 uM VitB12 recovering 24hrs), lane 5 (0.01 uM VitB12 treated for 24hrs), lane 6 (50 uM H2O2 + 0.01 uM VitB12 recovering 24hrs).

Series 2

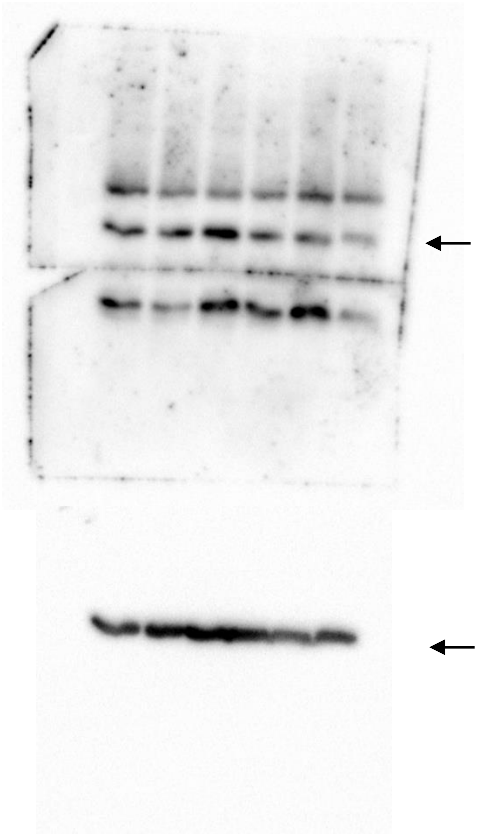

GAP-43 (the membrane was cut before incubation with the antibody).

ActinB (the membrane was cut before incubation with the antibody).

Lane 1 (Ctrl), lane 2 (50 uM H2O2 recovering 24hrs), lane 3 (1 uM VitB12 treated for 24hrs), lane 4 (50 uM H2O2 + 1 uM VitB12 recovering 24hrs), lane 5 (0.01 uM VitB12 treated for 24hrs), lane 6 (50 uM H2O2 + 0.01 uM VitB12 recovering 24hrs).

Series 3

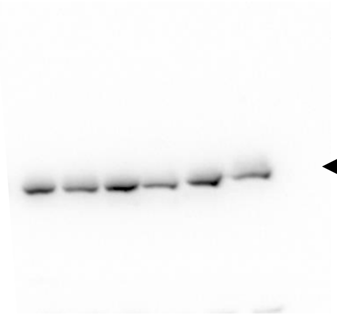

PARP1 (Low exposition) (the membrane was cut before incubation with the antibody).

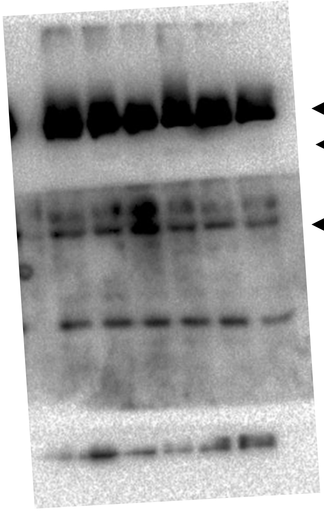

PARP1 (high exposition)

Cleaved PARP1 (the membrane was cut before incubation with the antibody).

Catalase (the membrane was cut before incubation with the antibody).

PCNA (the membrane was cut before incubation with the antibody).

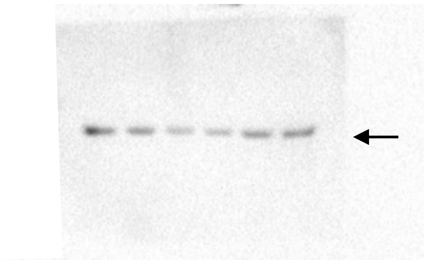

ActinB (the membrane was cut before incubation with the antibody).

Lane 1 (Ctrl), lane 2 (50 uM H2O2 recovering 24hrs), lane 3 (1 uM VitB12 treated for 24hrs), lane 4 (50 uM H2O2 + 1 uM VitB12 recovering 24hrs), lane 5 (0.01 uM VitB12 treated for 24hrs), lane 6 (50 uM H2O2 + 0.01 uM VitB12 recovering 24hrs).

Series 3

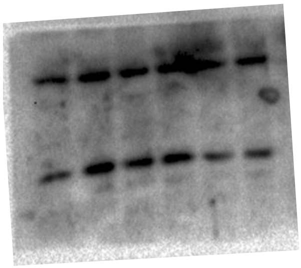

Catalase (the membrane was cut before incubation with the antibody).

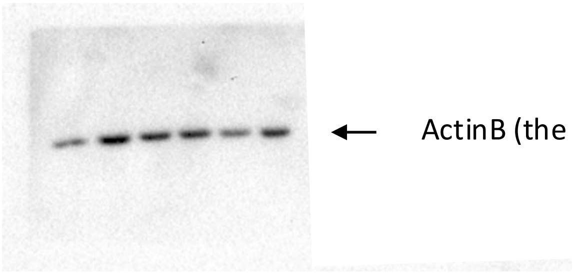

ActinB (the membrane was cut before incubation with the antibody).

Lane 1 (Ctrl), lane 2 (50 uM H2O2 recovering 2hrs), lane 3 (1 uM VitB12 treated for 2hrs), lane 4 (50 uM H2O2 + 1 uM VitB12 recovering 2hrs), lane 5 (0.01 uM VitB12 treated for 2hrs), lane 6 (50 uM H2O2 + 0.01 uM VitB12 recovering 2hrs).

Series 3

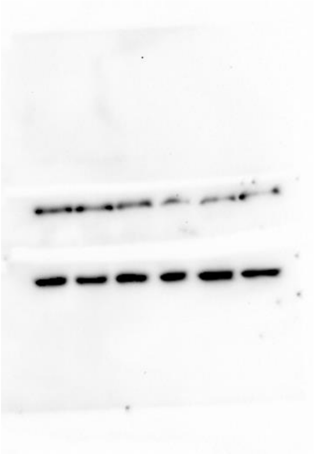

← GAP-43 (the membrane was cut before incubation with the antibody).

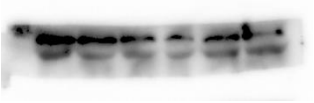

← ActinB (the membrane was cut before incubation with the antibody).

Lane 1 (Ctrl), lane 2 (50 uM H2O2 recovering 24hrs), lane 3 (1 uM VitB12 treated for 24hrs), lane 4 (50 uM H2O2 + 1 uM VitB12 recovering 24hrs), lane 5 (0.01 uM VitB12 treated for 24hrs), lane 6 (50 uM H2O2 + 0.01 uM VitB12 recovering 24hrs).

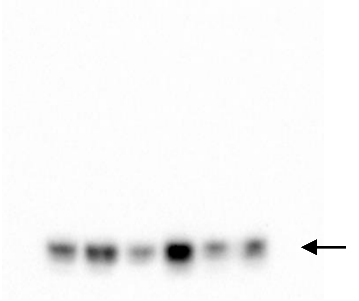

SYP (the membrane was cut before incubation with the antibody).

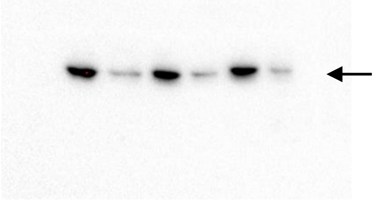

PCNA (the membrane was cut before incubation with the antibody).

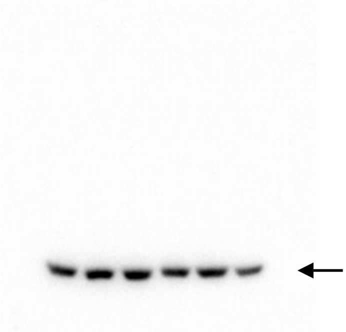

ActinB (the membrane was cut before incubation with the antibody).

Lane 1 (undifferentiated, series 1), lane 2 (differentiated, series 1), lane 3 (undifferentiated, series 2), lane 4 (differentiated, series 2), lane 5 (undifferentiated, series 3), lane 6 (differentiated, series 3),

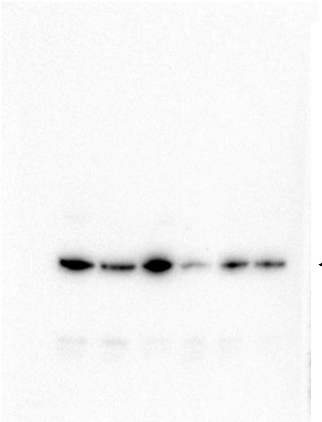

GAP-43 (the membrane was cut before incubation with the antibody).

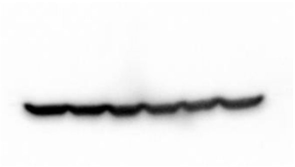

Actin (the membrane was cut before incubation with the antibody).

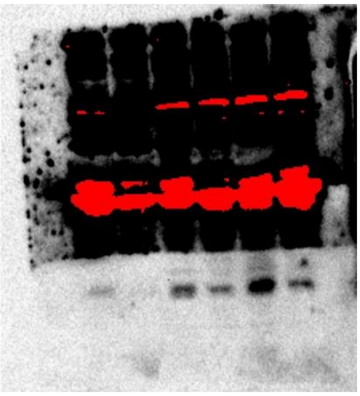

CD320 (the membrane was cut before incubation with the antibody).

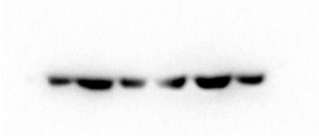

ActinB (the membrane was cut before incubation with the antibody).

Lane 1 (undifferentiated, series 1), lane 2 (differentiated, series 1), lane 3 (undifferentiated, series 2), lane 4 (differentiated, series 2), lane 5 (undifferentiated, series 3), lane 6 (differentiated, series 3),
